# Transverse depth-dependent changes in corneal collagen lamellar orientation and distribution

**DOI:** 10.1098/rsif.2014.0717

**Published:** 2015-03-06

**Authors:** Ahmed Abass, Sally Hayes, Nick White, Thomas Sorensen, Keith M. Meek

**Affiliations:** 1 Structural Biophysics Group, School of Optometry and Vision Sciences, Cardiff University, Maindy Road, Cardiff CF24 4HQ, UK; 2 Visual Science Bioimaging Labs (VSBL), School of Optometry and Vision Sciences, Cardiff University, Maindy Road, Cardiff CF24 4HQ, UK; 3 Diamond Light Source Ltd, Diamond House, Harwell Science and Innovation Campus, Didcot, Oxfordshire OX11 0DE, UK

**Keywords:** cornea, collagen, corneal biomechanics, X-ray scattering, collagen orientation

## Abstract

It is thought that corneal surface topography may be stabilized by the angular orientation of out-of plane lamellae that insert into the anterior limiting membrane. In this study, micro-focus X-ray scattering data were used to obtain quantitative information about lamellar inclination (with respect to the corneal surface) and the X-ray scatter intensity throughout the depth of the cornea from the centre to the temporal limbus. The average collagen inclination remained predominantly parallel to the tissue surface at all depths. However, in the central cornea, the spread of inclination angles was greatest in the anterior-most stroma (reflecting the increased lamellar interweaving in this region), and decreased with tissue depth; in the peripheral cornea inclination angles showed less variation throughout the tissue thickness. Inclination angles in the deeper stroma were generally higher in the peripheral cornea, suggesting the presence of more interweaving in the posterior stroma away from the central cornea. An increase in collagen X-ray scatter was identified in a region extending from the sclera anteriorly until about 2 mm from the corneal centre. This could arise from the presence of larger diameter fibrils, probably of scleral origin, which are known to exist in this region. Incorporation of this quantitative information into finite-element models will further improve the accuracy with which they can predict the biomechanical response of the cornea to pathology and refractive procedures.

## 1. Introduction

The structure of the cornea and surrounding limbus is such that it is able to maintain its shape under the forces applied by intraocular pressure, the cardiac cycle and the extraocular muscles during eye movement. As the material mechanical characteristics of the human cornea are highly dependent on its fibrillar collagen arrangement, many studies have been carried out to determine the precise orientation and distribution of collagen in the corneal stroma. Electron microscopy has shown that within the posterior stroma, collagenous lamellae (in which fibrils lie predominantly parallel to each other) appear to traverse the cornea from limbus-to-limbus, remaining in-plane, parallel to the corneal surface. However, in the anterior third of the stroma, many lamellae do not remain in-plane, as there is frequent branching and interweaving of lamellae [1,2]. Many (if not all) of the lamellae in the anterior stroma insert into the anterior limiting lamina (Bowman's membrane), intertwine with deeper fibres and reinsert back into Bowman's membrane to form bow spring-like structures [3]. Using the latest developments in nonlinear optical high-resolution microscopy, Winkler *et al.* [4] were able to quantify the angle of the out-of-plane collagen lamellae in the anterior 250 µm of the cornea. They showed that the range of lamellar angles relative to the stromal surface was highest in the anterior-most 83 µm of the corneal stroma and decreased with tissue depth. It has been suggested that the structural organization of the anterior cornea, which confers heightened elastic [3,5] and transverse shear moduli [6,7] and rigidity in extreme hydration [8], likely also plays a role in the maintenance of corneal curvature. This theory is further supported by observations of reduced lamellar interweaving and infrequent Bowman's membrane insertions in keratoconus (a pathology which affects the strength, and ultimately the shape of the cornea) [9].

Although, the arrangement of corneal collagen has been studied in detail by microscopy, the results have yielded little information about the large-scale quantitative orientation of collagen fibrils throughout the cornea as a whole. In recent years, this matter has largely been addressed through the use of synchrotron X-ray scattering. This technique allows structural, quantitative data to be rapidly obtained from full-thickness specimens, in a close-to-natural state (without the need for lengthy processing, such as associated with many microscopy techniques). Unlike most connective tissues, the cornea produces both a small-angle equatorial X-ray scatter pattern (caused by the uniformity of fibril diameters and the regular spacing of collagen) and a wide-angle equatorial pattern, arising from the lateral packing of the collagen molecules within the fibrils [10,11]. The intensity of the resulting X-ray scatter can provide quantitative information about the number of molecules (and hence relative mass density of collagen) lying in a given direction. In some of the earliest synchrotron studies on human corneas, small-angle X-ray scattering (SAXS) was used to demonstrate the significant amount of structural anisotropy in the centre of the cornea, where collagen was found to lie predominantly in the nasal–temporal and superior–inferior directions [12–14]. Later, analysis of wide-angle X-ray scattering (WAXS) data revealed the presence of a pseudo-annulus of collagen surrounding the cornea at the limbus [15] and in 2004, the first quantitative two-dimensional projected map of preferential lamellar orientation across the human cornea and limbus was published [16]. Subsequently, femtosecond laser technology was used to delaminate human corneas into anterior, mid and posterior stromal sections and thereby enable the predominant orientation of collagen in each region to be determined, thus highlighting the relative randomness of collagen orientation in the anterior stroma compared to the predominantly inferior–superior and nasal–temporal arrangement of collagen in the posterior stroma [17]. To date, most X-ray scattering studies have examined corneal tissue en-face, providing average measurements of collagen fibril orientation throughout the entire thickness of the cornea; however, the relatively recent development of micro-focus X-ray beams has provided new opportunities for examining sagittal sections of the cornea ‘edge-on’, to obtain detailed depth profiled information on lamellar structure. The feasibility of this technique was demonstrated by Quantock *et al.* [18] but due to the examined corneas being well above physiological hydration it was not possible to ascertain accurate information regarding the extent and direction of lamellar branching that likely occurs *in vivo*.

The aim of this study was to obtain micro-focus WAXS data at fine intervals throughout the entire thickness of sagittal sections taken from physiologically hydrated human corneas (perfusion-fixed under pressure) and use this information to generate an accurate numerical representation of lamellar branching as a function of tissue depth from the centre of the cornea to the temporal limbus.

## 2. Material and methods

### 2.1. Sample details

Six eyes from five donors supplied by the National Disease Research Interchange (Philadelphia, PA, USA) were enucleated, frozen and transported at −80°C to Cardiff University. These eyes were thawed prior to experimentation. A further eye was obtained from the Bristol Eye Bank and transported chilled to Cardiff University (table 1).

**Table 1.** Sample information and measurements of central corneal thickness (CCT).

sample	right/left	sex	age (years)	time between death and enucleation (hours : minutes)	CCT on arrival (µm)	stromal thickness during X-ray data collection (µm)
1L	L	F	88	07 : 00	920	580
2L^a^	L	F	72	06 : 05	690	600
2R^a^	R	F	72	06 : 05	810	580
3L	L	M	70	07 : 00	710	540
4R	R	M	61	03 : 00	810	600
5L	L	M	63	10 : 09	720	580
6L	L	F	72	24 : 05	760	500

^a^Left/right pair of eyes from the same donor.

### 2.2. Specimen preparation

**Figure 1.**
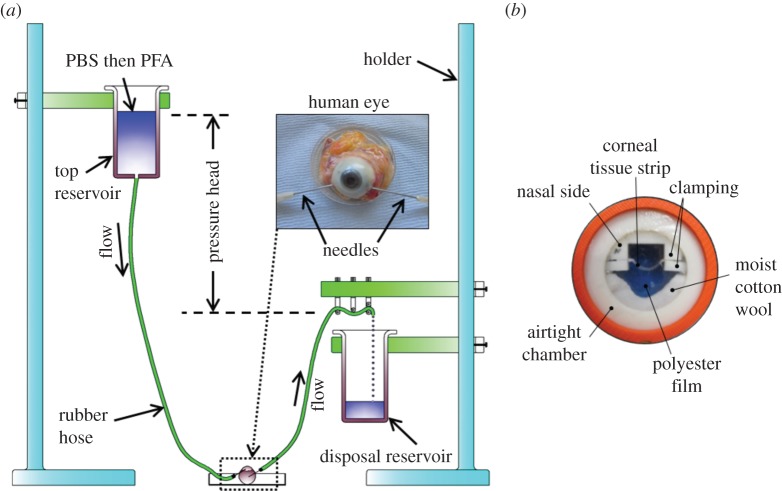
The main stages of sample preparation involved de-swelling the cornea and fixing the eye under pressure (*a*), followed by the mounting of a 1 mm wide sagittal corneal strip in an airtight clamping chamber (*b*). The photograph in (*b*) was taken on a blue background in order to visualize the corneal strip within the chamber.

Prior to fixation, the central corneal thickness was measured using a Pachette2 Ultrasonic Pachymeter (DGH Technology, USA) (table 1). To counteract the effects of post-mortem swelling, the corneas were thinned prior to fixation, using a modification of the phosphate-buffered saline (PBS) infusion technique described previously by Winkler *et al.* [4]. A pressure head of PBS was created by holding a reservoir 40 cm above the eye and inserting a needle at the end of the hose through the sclera and into the anterior chamber (figure 1*a*). A pressure of 20 mmHg was maintained for 2 min before a second needle (connected to a disposal reservoir) was inserted into the anterior chamber. By adjusting the pressure head to 23 cm, the eyeball was kept under pressure at 17 mmHg for 30 min. The PBS in the top reservoir was then gradually replaced with 4% paraformaldehyde (PFA) in PBS and allowed to perfuse for 5 min, thus enabling the eye to be fixed at close to physiological pressure (12–22 mmHg). During the fixation process, corneal surface hydration was maintained by regular application of PBS (one drop every 5 min). On completion, the eye was submerged in 4% PFA and stored at 4°C for 3 days until required for data collection.

Immediately prior to data collection, the cornea, with a 3 mm wide scleral rim (marked at the nasal position with a surgical skin marker pen), was removed from each eye. The corneal epithelium was carefully removed using a scalpel blade. Corneas with an average stromal thickness of 569 µm were achieved (table 1). A 1 mm wide sagittal strip was then cut through the centre of each cornea (along the nasal–temporal meridian) using a specially designed strip-cutter (consisting of two parallel-mounted razor blades), and these strips were used for X-ray scattering. A second strip was cut from one cornea and set aside for second harmonic generation (SHG) nonlinear microscopy (see below). In order to maintain the natural curvature of the cornea during X-ray data collection, each strip was clamped at its scleral edges and placed inside a custom-made airtight chamber, sealed with polyester films (Mylar; DuPont-Teijin Films, Middlesbrough, UK) (figure 1*b*). The chamber was mounted in a motorized goniometer stage which was used, together with an in-line microscope camera (modified Fetura system from Qioptiq, Munich, Germany), to ensure that the X-ray beam was parallel to the top and bottom surfaces of the strip. The alignment was ensured by adjusting the goniometer stage angle electronically until satisfactory alignment was seen through the in-line microscope camera. The potential for tissue dehydration during X-ray exposure was minimized by placing a piece of moist cotton wool within the chamber alongside the corneal strip and monitoring the thickness of the strip before and after X-ray exposure by means of the in-line camera.

### 2.3. Second harmonic generation microscopy

A 160 µm wide nasal-to-temporal section was cut from one of the strips from sample 3L using a sledge microtome (HM440E; Microm, Walldorf, Germany). The section was then immediately covered by 1 : 1 PBS glycerol solution and sealed between a 1 mm microscope glass slide and a 0.16 mm coverslip. SHG images were acquired with an LSM 510 META NLO instrument (Carl Zeiss, Cambridge, UK) and 20× 0.8NA Plan Apo objective. Illumination was typically about 3 mW in approximately 140 fs pulses at 800 nm from a Chameleon Ultrafast laser (Coherent Scotland Ltd, Glasgow, UK). SHG contrast images were collected in the forward propagating direction at 400 ± 10 nm through a high-extinction near-infrared blocking filter HQ400/20m-2p (Chroma Technology Corp., VT. USA). Representative optical sections through the physical corneal slices, away from the cut surfaces, were collected using a simple tiling procedure with an automated stage but without any image processing, edge adjustments or other data manipulation. The pixel spacing in the SHG images was 0.77 µm.

### 2.4. X-ray data collection

**Figure 2.**
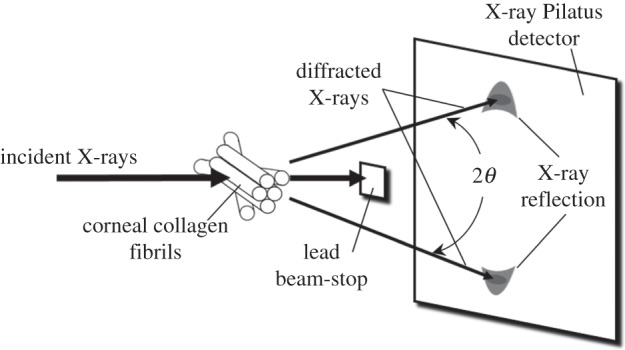
Generation of the wide-angle corneal X-ray scatter pattern. The plane of the corneal tissue is regarded as being the *x*–*y* direction and the X-rays are applied in the out-of-plane *z*-direction.

**Figure 3.**
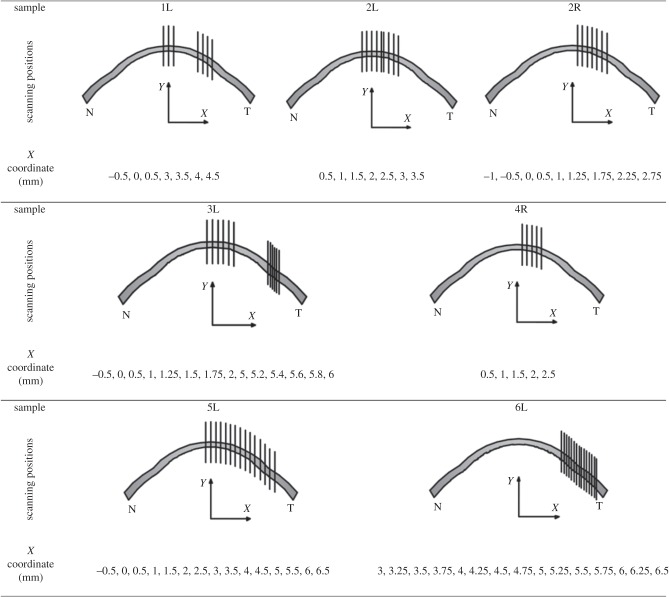
Schematic showing the X-ray scan positions for each sample. The geometric centre of the spherical central cornea corresponds to the origin of the coordinate system shown in table 2.

WAXS experiments were carried out on beam-line IO2 at the UK's national synchrotron facility, Diamond Light Source (Didcot, UK). X-ray scatter patterns were collected on a vacuum-compatible Pilatus detector placed 350 mm behind the specimen (figure 2); a lead beam stop positioned between the specimen and the detector was used to prevent any undiffracted X-rays from damaging the detector sensitive area. X-ray reflections occur symmetrically on the X-ray pattern at a scattering angle of *θ* with respect to the incident direction. Using a 40 µm wide by 20 µm high micro-focus X-ray beam with a wavelength of 1.0 Å, X-ray scatter patterns resulting from a 0.5 s X-ray exposure were collected at 20 µm intervals throughout the entire thickness of the corneal stroma. As illustrated in figure 3 and table 2, multiple vertical scans were performed at central, peripheral and limbal locations. The resulting X-ray scatter patterns were processed using MATLAB software (Mathsworks, USA) and calibrated against the 0.304 nm reflection of powdered calcite [10]. Collagen scatter intensity in each pattern was adjusted to account for the micro-focus X-ray beam dimensions, exposure time and Diamond's storage ring current during each experiment.

**Table 2.** X-ray scan positions in the cornea, limbus and sclera (shown as *x*-coordinates).

sample	*x*-coordinate for X-ray scan positions																														
	central cornea									peripheral cornea												limbus/sclera									
**X (mm)**	−1	−0.5	0	0.5	1	1.25	1.5	1.75	2	2.25	2.5	2.75	3	3.25	3.5	3.75	4	4.25	4.5	4.75	5	5.2	5.25	5.4	5.5	5.6	5.75	5.8	6	6.25	6.5
**1L**		▪	▪	▪									▪		▪		▪		▪												
**2L**				▪	▪		▪		▪		▪		▪		▪																
**2R**	▪	▪	▪	▪	▪	▪		▪		▪		▪																			
**3L**		▪	▪	▪	▪	▪	▪	▪	▪												▪	▪		▪		▪		▪	▪		
**4R**				▪	▪		▪		▪		▪																				
**5L**		▪	▪	▪	▪		▪		▪		▪		▪		▪		▪		▪		▪				▪				▪		▪
**6L**													▪	▪	▪	▪	▪	▪	▪	▪	▪		▪		▪		▪		▪	▪	▪

### 2.5. X-ray diffraction analysis

**Figure 4.**
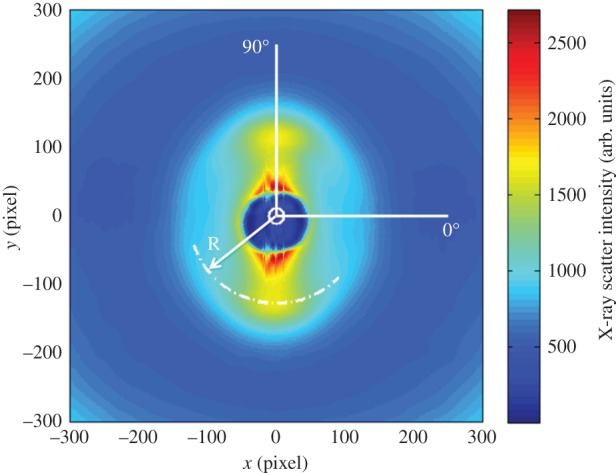
An X-ray pattern generated from corneal tissue has a collagen diffraction peak centred at radius R. The intensity of X-ray scatter shows angular dependence, with more scatter along 90° than along 0° in this example.

**Figure 5.**
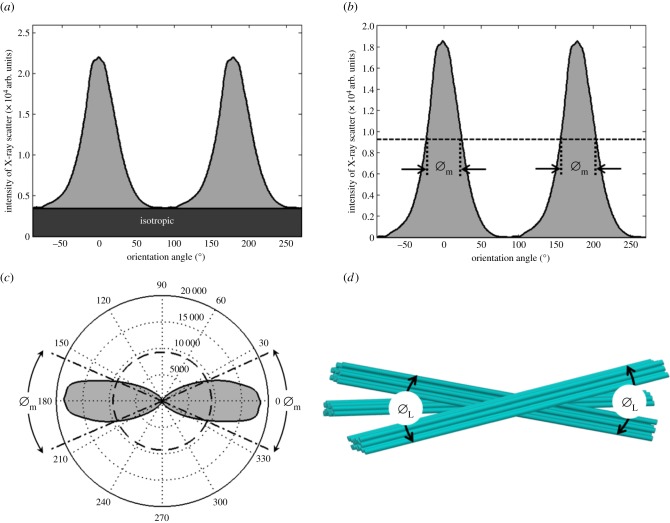
The total collagen scatter intensity consists of an isotropic component arising from collagen fibrils running through the thickness of the corneal strip, and a non-isotropic component from fibrils at different depths in the plane of the strip (*a*). The angular spread of collagen molecules Ø_m_ is measured as the width of the aligned collagen WAXS reflection at half of its peak height (shown as a dashed line) (*b*). For display purposes, the predominant direction of the collagen molecules and Ø_m_ may be shown as a polar plot (*c*). The physical interpretation of collagen lamella spread angle Ø_L_ (which is calculated directly from Ø_m_) is shown schematically in *d*. (Online version in colour.)

At a given point in the tissue, collagen orientation is not isotropic, so the total scattering distribution has an angular dependence. For example, in the scatter pattern shown in figure 4, scattering along the 0° direction is significantly lower than along 90°. The relative collagen orientations in different directions were determined from each scatter pattern as described in detail previously [10,19] except that here we increased the resolution by making 720 radial divisions over 360° rather than 256. The total collagen scatter intensity distribution was shifted by 90° to account for the fact that equatorial scatter occurs at right angles to the collagen axis [10] (figure 5*a*). The total collagen scatter intensity comprises isotropic and non-isotropic components. Unlike in our previous papers [10], which have examined the cornea en-face, the isotropic scatter in this case arises from collagen fibrils running through the thickness of the cut strip. This itself will contain two contributions, one taking the form of a uniform ring, which arises from a sub-population of collagen fibrils that are aligned with the direction of the incident X-ray beam and another from lamellae running through the strip obliquely (i.e. not parallel to the X-ray beam), which will contribute at different angles to the isotropic scatter. The non-isotropic scatter arises predominantly from collagen within the section plane. So long as hydration is constant and any induced changes in hydration during sample preparation are uniform in the tissue, the total X-ray scatter intensity (both isotropic and non-isotropic) will be a reasonable representation of the mass density of the collagen sampled by the beam, so can be used to approximate the variation of mass density across the samples.

The isotropic scatter was subtracted out in the collagen molecular spread angle computation (figure 5*b*) because of the unknown contribution from the collagen fibrils running parallel to the X-ray beam. The collagen molecular spread angle Ø_m_ was measured as the width of the non-isotropic collagen scatter peak at half of its peak height as can be seen in figure 5*b,c*. However, because collagen molecules are not parallel to the fibril axis [20] the angular spread of fibrils and hence, also lamellae (Ø_L_), is less than the measured angular spread of the molecules from which they are constituted (Ø_m_) (figure 5*d*). The correction factor (*k* = 0.385) may be found by calculating the ratio of the width at half normalized height from the wide-angle corneal diffraction pattern (arising from molecular scatter) and the same parameter from the small-angle diffraction pattern (arising from fibril scatter) from the same specimen [10]. The angular spread of collagen molecules is represented as a polar plot in figure 5*c*, and the physical interpretation of the polar plot in figure 5*c* is shown in figure 5*d* after correcting Ø_m_ to Ø_L_, where Ø_L_ = *k* Ø_m_. The average amount by which lamellae deviate from their predominant direction is therefore represented by ±Ø_L_/2 and will be referred to as the lamellar inclination.

## 3. Results

**Figure 6.**
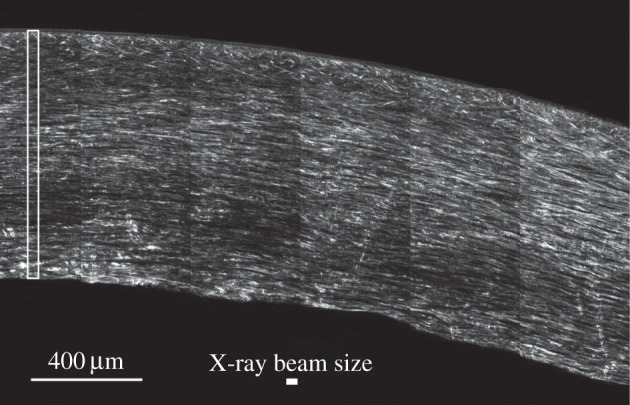
SHG image of an optical section taken about 100 µm below the cut surface of the corneal physical section. An area (cropped from a larger image) of approximately 6 × 4 images each 512 × 512 pixels in size is shown, using simple tiling without orientation correction, edge matching or any image processing. Bright features correspond to SHG scattering at 400 nm, in the forward (transmitted) direction, of laser light at 800 nm in approximately 140 fs pulses. SHG contrast in the image derives from a complex distribution of nonlinear susceptibility in non-centro-symmetric fibrillar collagen structures of varying diameters as well as linear scattering at both the primary and secondary wavelengths. The cross-sectional area of the microfocus X-ray beam is shown by a solid white rectangle and the cross-sectional area of a typical scan is shown by an open white rectangle.

Representative optical sections, comprising adjacent images each of 512 × 512 pixels concatenated into a single picture (figure 6), show the SHG contrast obtained from the non-centro-symmetric fibrillar collagen structures. Even without any image processing, the differences previously described between anterior and posterior stroma [3] are evident, with many out-of-plane lamellae in the anterior stroma gradually transforming to lamellae parallel to the anterior surface in the posterior stroma. Figure 6 also shows the volume of tissue examined in a single X-ray scan, together with the size of the X-ray beam. This gives an idea of the cross-sectional area of the tissue that was sampled in each X-ray pattern.

The WAXS scatter patterns contain information about the molecular spacing between collagen molecules within fibrils, as well as information about fibril inclination angles [10]. We found that the intermolecular Bragg spacing, averaged as a function of tissue depth, remained within the range 1.64–1.69 nm in all samples at all positions across the cornea. This suggests that the hydration of the fibrils themselves is not a function of radial position in the cornea.

**Figure 7.**
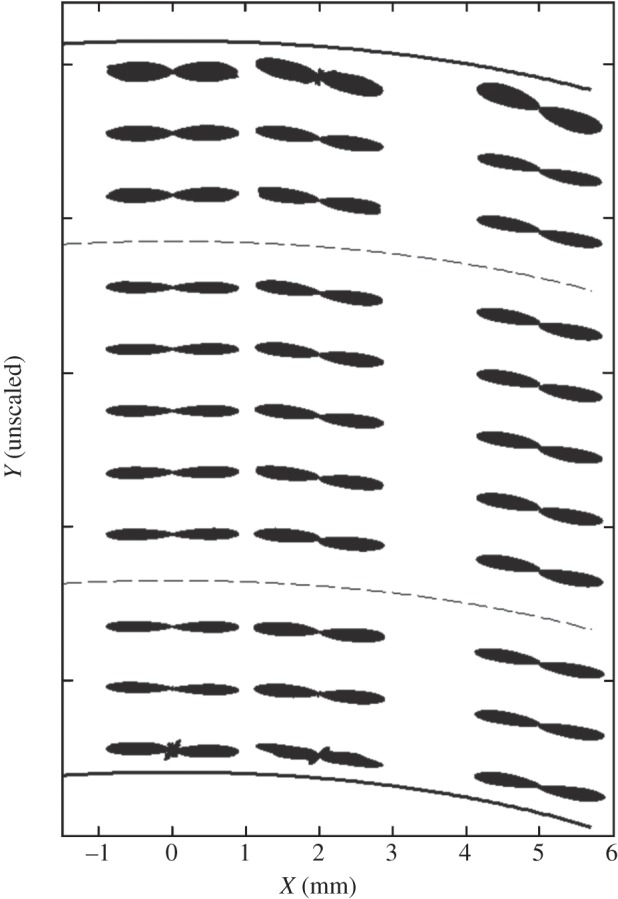
Polar plots depict the predominant orientation of collagen in the anterior, mid and posterior stroma of the central and peripheral regions of the human cornea (specimen 3L). For display purposes, only selected polar plots from each stromal depth have been shown and a broken line has been used to separate the anterior, mid and posterior stroma. Owing to variations in collagen X-ray scatter intensity with tissue depth, the polar plots have been normalized.

Figure 7 shows polar plots generated from X-ray data collected in the anterior, mid and posterior stroma at three radial positions in cornea 3L. The plots show that the average course of lamellae remains parallel to the tissue surface across, and throughout the depth of, the tissue. However, anterior-most plot(s), seen at the top, are ‘fatter’ than those elsewhere, depicting the increased spread of inclinations of lamellae with respect to the tissue surface, which is shown in greater detail in figures >8 and >9. To test whether this spread of inclination angles follows a normal distribution, representative plots from different depths were analysed using MATLAB software. Statistical analysis with Lilliefors and Kolmogorov–Smirnov tests showed that, at all depths, the lamella inclination angles follow Gaussian distributions.

**Figure 8.**
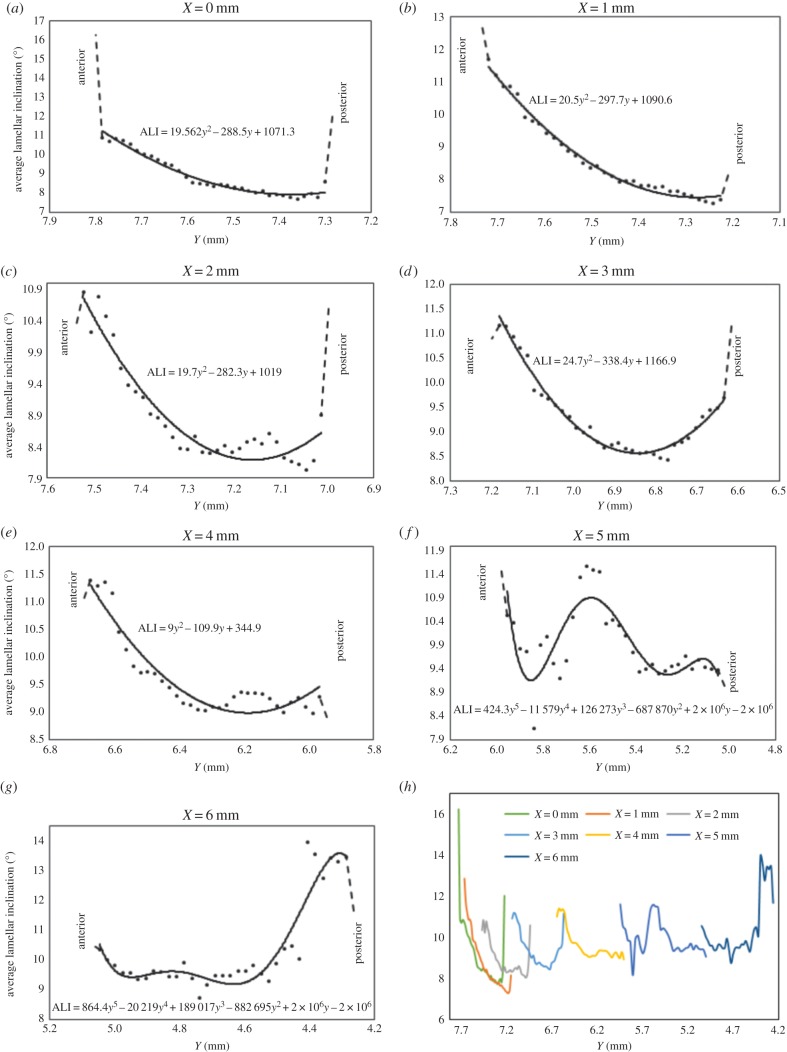
Averaged lamellar inclination (ALI) as a function of tissue depth at specific radial positions across the central cornea (*a–c*) and temporal aspect of the peripheral cornea (*d,e*), limbus (*f*) and near sclera (*g*). Owing to possible edge effects in the first and last data points, these parts of the curves are shown by broken lines. Data from (*a–g*) are combined in (*h*) to allow a quantitative comparison of lamellar inclination as a function of tissue depth at the different positions across the cornea into the adjacent temporal sclera.

**Figure 9.**
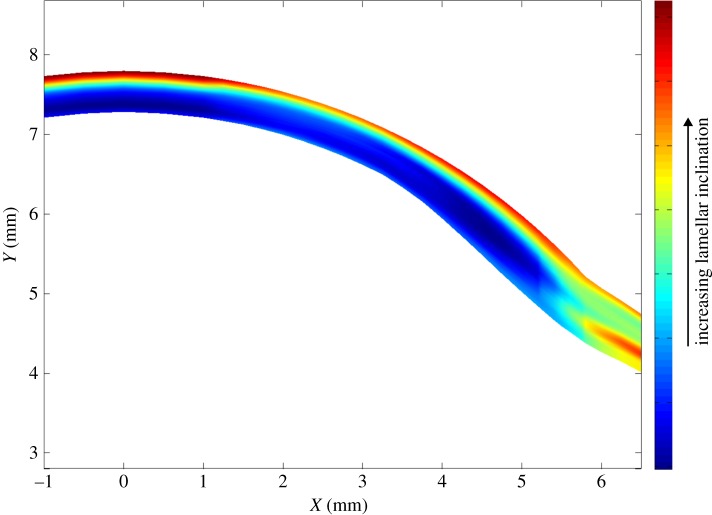
Averaged lamellar inclination (in degrees) across the central and temporal aspect of the cornea, limbus and adjacent sclera.

Figure 8 shows the averaged variation in lamellar inclination as a function of tissue depth at different radial positions across the central (*a–c*) and peripheral cornea (*d,e*), limbal region (*f*) and adjacent sclera (*g*). It should be noted that, except at the optical centre of the cornea, these represent oblique scans through the cornea; in other words, as depicted in figure 3, not all scans run perpendicular to the corneal surface. In the prepupilliary region of the cornea (figure 8*a–c*), lamellar inclinations of up to 16° are observed in the anterior stroma; the reader should be reminded however, that this value represents the average of a distribution of angles at each sampled position and not the maximal inclination of lamellae at a given position. This angle diminishes with depth and levels off at 7.5–8.5° mid-way through the tissue. There is evidence of an increase in lamellar inclination in the posterior layers of the stroma in the central and peripheral cornea (up to a 4 mm radius) (figure 8*a–e*). Near the limbus (figure 8*f*), in the mid-depth of the stroma inclination angles increase and then decrease again. In the near sclera (figure 8*g*), there is a pronounced increase in the inclination angle within the posterior layers. The graphs in figure 8*a–g* are combined in figure 8*h* to allow a quantitative comparison of the variation in lamellar inclination as a function of depth at the different positions across the cornea. Across the corneal surface, lamellae insert into Bowman's layer at angles averaging between 10.8° and 16°. Away from the optical centre of the cornea deeper stromal lamellae gradually become more interwoven (greater lamellar inclination).

In figure 9, the average values of lamellar inclination from all samples at all positions are plotted after robust smoothing of the data [21]. This figure highlights that, while the cornea contains interwoven lamellae only in the anterior layers, the limbal and scleral lamellae are interwoven at all depths (see tables A, B and C in the electronic supplementary material).

**Figure 10.**
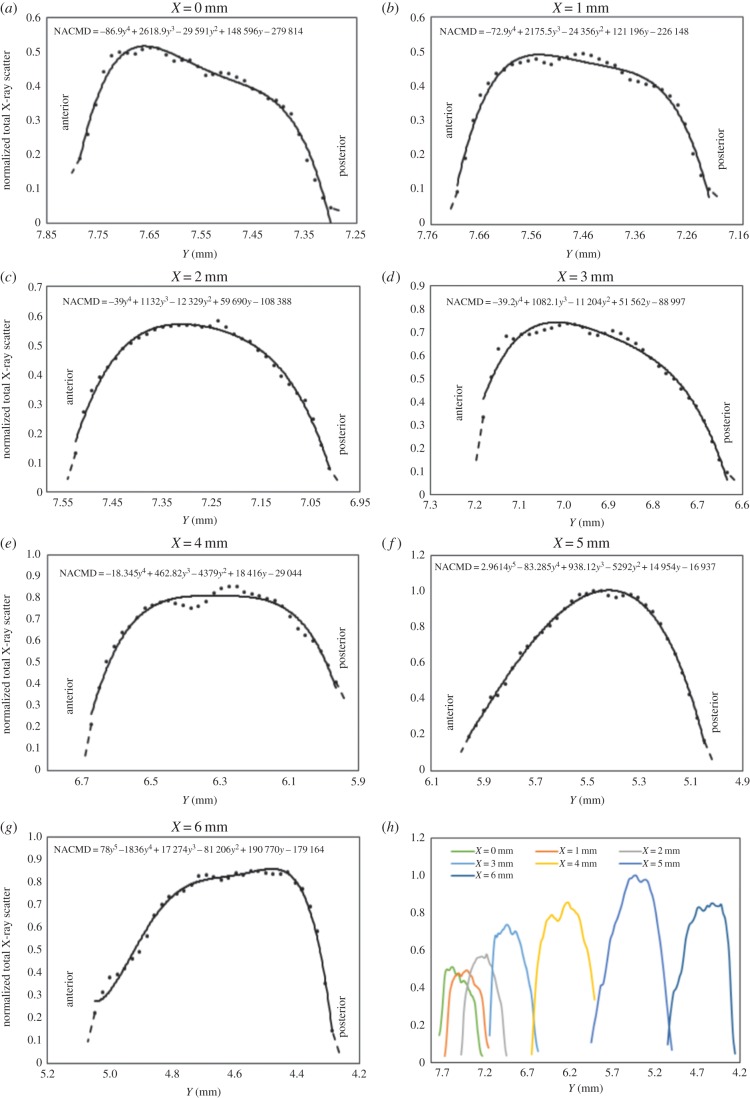
Normalized average total X-ray scatter intensity (representing the normalized average corneal mass density, NACMD) shown as a function of tissue depth at specific radial positions across the central cornea (*a–c*) and temporal aspect of the peripheral cornea (*d,e*), limbus (*f*) and near sclera (*g*). The total X-ray scatter at each position in an individual scan was normalized against the maximum value of all the scans shown. Owing to possible edge effects in the first and last data points, these parts of the curves are shown by broken lines. Data from (*a–g*) are combined in (*h*) to allow a quantitative comparison of the normalized total X-ray scatter as a function of tissue depth at the different positions across the cornea and limbus.

**Figure 11.**
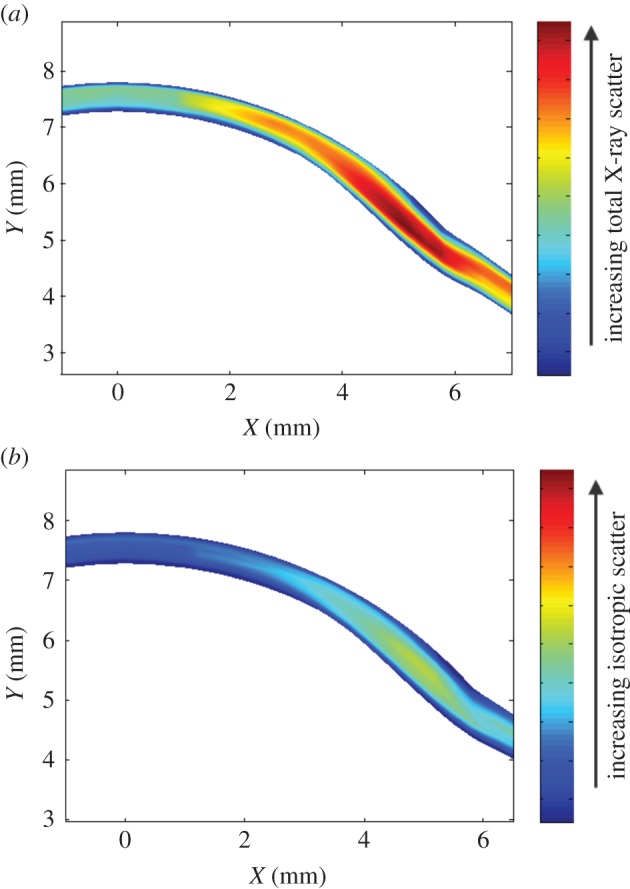
Contour plots showing the average total (*a*) and isotropic (*b*) X-ray scatter intensity across the central and temporal aspect of the cornea and limbus.

The variation in the total X-ray scatter intensity as a function of tissue depth is shown in figure 10. At the optical centre of the cornea (figure 10*a*), after an initial increase, there is a steady reduction in the X-ray scatter intensity as a function of tissue depth followed by a rapid decrease in the posterior stroma. A similar trend occurs at positions away from the centre (figure 10*b–d*) but in the peripheral cornea, limbus and sclera (figure 10*e–g*) the trend changes slightly towards a gradual increase in X-ray scatter intensity with depth followed by the same rapid drop in the most posterior layers. The relative scale of these effects can be seen numerically in figure 10*h* (see tables A, B and D in the electronic supplementary material), and also in figure 11*a*, where smoothed data from all the scans are shown. It is evident from figure 11*a* that there is a region of intense total X-ray scatter, originating in the sclera and extending into the peripheral cornea. Figure 11*b* shows just the isotropic scatter contribution (figure 5) which arises from lamellae running through the section thickness; in real terms, these lamellae can be envisaged as extending out of the page. This shows a strong feature centred near the limbus about midway between the anterior and posterior surface. Comparison of the relative intensities of the unsmoothed data used to produce figure 11*a,b* indicates that the contribution of the isotropic scatter to the total scatter is approximately 50% across the cornea and limbus.

## 4. Discussion

In this paper, we have, for the first time, used X-ray scattering techniques to quantify the lamellae inclination angles and X-ray scatter intensity through the thickness of the corneal stroma. Because a whole human donor eye was required for each sample strip, it was not possible to investigate these distributions along other meridians. Nevertheless, the results obtained are an important first step to elucidating the full three-dimensional organization of stromal lamellae.

In previous studies, we have used WAXS to determine the preferred lamellar orientations throughout the cornea and sclera [16,17,19] on the assumption that the average direction of the molecules aligns with the average direction of the fibrils which they constitute. This has been a reasonable approximation since we were primarily concerned with quantifying the relative variation of mean preferred lamellar orientations across the cornea. However, it is a significant problem when quantifying the angular spread of lamella inclination angles as carried out in the current work, and the molecular angular spread measurements therefore needed to be corrected by a factor of about 60%. In theory, SAXS (which arises from the fibrils themselves) would overcome the need for such a correction. However, this technique is also subject to significant limitations such as its sensitivity to small variations in tissue hydration and the influence of non-collagenous molecules associated with the collagen fibrils. Since WAXS reflections are relatively insensitive to changes in tissue hydration our use of WAXS should provide a better estimate of the variation in collagen mass density across the tissue.

WAXS is a unique and well-established method for measuring the lateral spacing between individual fibril-forming collagen molecules. As collagen intermolecular spacing is known to be influenced by both the hydration of the fibrils [22,23] and by the degree of molecular cross-linking [24], the absence of any major changes in intermolecular spacing with tissue depth or radial distance from the corneal centre, suggests that the collagen molecular arrangement within the fibrils is uniform throughout the cornea. This finding is consistent with previous SAXS data which found there to be no significant depth-dependent change in the average diameter of corneal collagen fibrils [18]. The intermolecular spacing measurements obtained in this study were similar to those reported in previous X-ray scattering studies performed on full-tissue thickness, PFA-fixed human corneas [25] and ‘fresh’ human corneas [26].

**Figure 12.**
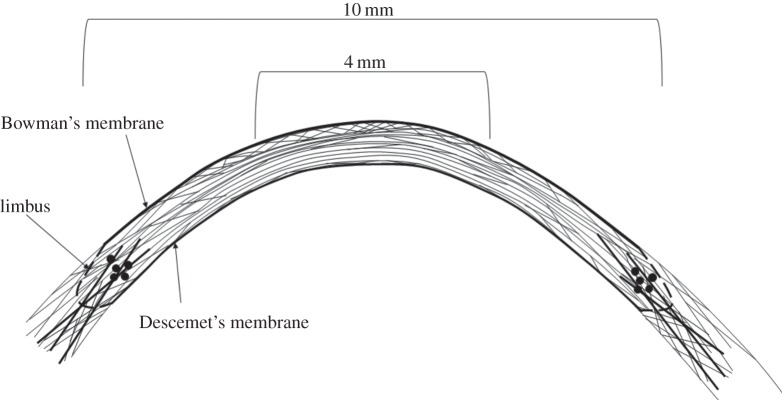
Schematic of the proposed arrangement of collagen lamella in a cross-section of the corneal stroma. In the central cornea, anterior lamellae make relatively large angles with Bowman's membrane. These angles decrease in the deeper stroma then increase slightly in the posterior-most lamellae. In the peripheral cornea between 8 and 10 mm, lamellar interweaving extends throughout the whole thickness of the tissue. In the peripheral stroma and limbus, anchoring lamellae (probably of scleral origin) reinforce the tissue in the mid-posterior layers (dark lines) without entering the central stroma. Solid black circles are used to represent lamellae cut in cross-section at the postulated position of the limbal pseudo-annulus.

Since the natural curvature of the cornea was also maintained throughout the procedure (as a result of it being perfusion-fixed under pressure prior to data collection), the reported measurements of lamellar inclination as a function of tissue position and depth may therefore be seen to reasonably represent the *in vivo* arrangement of lamellae within the human cornea. This arrangement is illustrated schematically in figure 12.

Using this technique, we have shown that collagen lies predominantly parallel to the corneal surface at all tissue depths but an increase in lamellar inclination occurs in the anterior stroma and also, to a lesser extent, in the final 20–30 μm of the posterior stroma. The measured increase in lamellar inclination in the anterior cornea clearly reflects the increased lamellar interweaving seen in this region [1,2] and the presence of large quantities of transverse fibres which connect one or many adjacent layers, and frequently insert into Bowman's membrane [4]. The complex arrangement of collagen in the anterior stroma has been further demonstrated by X-ray scattering studies which revealed an isotropic arrangement of lamellae within the plane of the cornea as viewed en-face [17]. The precise arrangement of collagen in the anterior cornea, which has been shown to confer additional mechanical strength [3,5–7] and rigidity in extreme hydration [8], is believed to play an important role in the biomechanics of the tissue by resisting intraocular pressure and maintaining correct curvature. In the anterior cornea, the greatest inclination angles appear to occur within the central cornea and are reduced in the rest of the cornea and sclera. This supports the idea that lamellar interlacing is involved in the maintenance of corneal curvature, since the curvature of the centre is greater than that of the periphery [27].

It was noted in figure 8 that the inclination angle does not appear to reduce to zero at any depth or radial position in the cornea but remains greater than 7.2°. This is may be due to the presence of some residual crimp which exists in eyes fixed at close to physiological intraocular pressure. In whole eye *ex-vivo* experiments, it has been shown that crimp is present under normal intraocular pressure loading [28]. This may be of some significance as the mechanical behaviour of the cornea is closely correlated with its crimping morphology [29].

The quantitative information derived from this study concurs with previous knowledge of the cornea obtained from electron microscopy. In the posterior stroma, lamellae are seen to run continuously from limbus to limbus and are stacked parallel to the corneal surface, with branching occurring predominantly within the plane of the cornea [1,2,30]. En face X-ray scattering studies of human cornea have shown that within this region collagen lies predominantly in the vertical and horizontal meridians (directed towards the four major rectus muscles). It is thought that the arrangement of collagen in the posterior stroma may help to distribute strain in the cornea by allowing it to withstand the pull of the extraocular muscles [17].

The observed increase in lamellar inclination observed in the final 20–30 μm of the posterior stroma may be related to the presence of increased residual crimp angles in these lamellae. In the pre-Descemet's stromal layer [31], there are also electron microscope observations of a very thin sheet of thin randomly arranged, interwoven fibrils embedded in the amorphous substance of Descemet's membrane [1,32]. Unlike the anterior limiting lamina [33], the pre-Descemet's layer does not adhere strongly to the stroma, allowing it to be dissected surgically as a sheet [34,35] and detached from the rest of the stroma without rupturing [35,36]. However, it should be borne in mind that the current results from the most posterior lamellae are approaching the limit of resolution of our data (20 µm through the corneal thickness), so they should be regarded with a degree of caution at this stage.

On the assumption that the variation of total X-ray scatter is directly related to the variation in the mass density, it would appear from figure 10 that collagen mass density is reduced near the anterior and posterior surfaces of the cornea. At the centre of the cornea (figure 10*a*), after the initial increase there is a gradual, then a steeper reduction in mass density. This is likely due to the fact that the posterior stroma contains different hydrophilic glycosaminoglycans and is therefore more hydrated than the anterior stroma [37] so the collagen fibrils are further apart [38].

Figure 11*a* shows the presence of a large streak of increased total collagen mass density apparently originating in the sclera. We speculate that this arises from the presence of larger diameter fibrils, probably of scleral origin, which are known to exist in this region. We have termed these structures ‘anchoring lamellae’ [16]. Some evidence for the existence of these structures has also been reported using nonlinear microscopy [3]. Figure 11*b* shows a distinct structure near the limbus that arises from lamellae cut in cross-section. This occurs at the same position as the limbal pseudo-annulus, a structure composed of collagen and mature elastic fibres [39] that is supposed to help sustain the change in curvature between the cornea and the surrounding sclera [40].

The only previous attempt to quantify lamellar inclination angle was carried out by Winkler *et al.* [3,4] using nonlinear SHG imaging. Our current findings are in accordance with Winkler *et al.* [4], also showing lamellar inclination to decrease with increasing stromal depth; however, our study goes further, making use of recent advances in microfocus X-ray data collection to obtain quantitative information about lamellar inclination throughout the entire stromal thickness of seven human corneas. In the anterior 250 μm of the stroma, Winkler *et al.* found that the inclination angles followed a Gaussian distribution centred at 0° (i.e. parallel to the corneal surface). This is in accord with our own study which found Gaussian distributions at all depths in the stroma. As with Winkler *et al.* we also measured the width at half maximum as a measure of the spread of inclination angles at a given depth. At the centre of the cornea, Winkler *et al.* found inclination angles (half their presented angular distribution width) of about 7° at the anterior surface. From figure 8*a*, it can be seen that we found angles more than 11° at this position (even if possible edge effects in our data are ignored). One difference between the studies is that we only examined angles within the central–temporal cross-section, whereas they examined angles in all four quadrants of the cornea. Also, we fixed the eyes at physiological intraocular pressure whereas they used an elevated pressure. Finally, Winkler *et al.* found differences in the depth dependence of the inclination angle that were position dependent. We also found such a gradient throughout the whole cornea, which was greatest near the centre of the cornea (figure 8*a,b*), but there was a very different dependence of inclination angle on tissue depth in the limbus and sclera (figure 8*f,g*).

As the mechanical behaviour of corneal stroma is quite complex [41,42], geometric and material characteristics guesstimates are generally required in finite-element modelling analysis because of either the lack of scientific knowledge or the limited performance of the software and computing system used. Most recent finite-element models of corneal biomechanical behaviour regard the tissue as being anisotropic and having strongly directional mechanical properties [43]. The models are typically based on inputted data from X-ray scattering studies which show that collagen is orientated in all directions but preferentially aligned along the superior–inferior and nasal–temporal directions [42,44,45]. The accuracy of these models is however limited by the lack of numerical information available to modellers regarding the structural variations that occur as a function of tissue depth. An attempt to incorporate depth-dependent variations in the inclination of lamellae was recently made by Petsche & Pinsky [46], using measurements taken from the second harmonic-generated images described above. The authors concluded that consideration of lamellar inclination was crucial for accurate modelling of different modes of mechanical deformation [46]. It is anticipated that inclusion of the quantitative information generated from this study, characterizing the variations in lamellar inclination and collagen mass distribution as a function of tissue depth, will lead to further improvements in the accuracy of computational models aimed at predicting corneal biomechanical behaviour.

## Supplementary Material

Averaged lamellar inclination and normalised average total x-ray scatter intensity across the central and temporal aspect of the cornea and limbus
