# Supplementary material for: Transverse depth-dependent changes in corneal collagen lamellar orientation and distribution
Source: J R Soc Interface. 2015 Mar 6;12(104):20140717. doi: 10.1098/rsif.2014.0717 (PMC4345466; doi:10.1098/rsif.2014.0717)
Supplement: Averaged lamellar inclination and normalised average total x-ray scatter intensity across the central and temporal aspect of the cornea and limbus [file rsif20140717supp1.doc]

**Supplementary material for the paper:**

**Transverse depth-dependent changes in corneal collagen lamellar orientation and distribution**

Ahmed Abass^1^, Sally Hayes^1^, Nick White^1^, Thomas Sorensen^2^ and Keith M Meek^1*^

^1^Structural Biophysics Group, School of Optometry and Vision Sciences, Cardiff University, Maindy Road, Cardiff CF24 4HQ, UK

^2^Diamond Light Source Ltd, Diamond House, Harwell Science and Innovation Campus, Didcot, Oxfordshire, OX11 0DE, UK

^*^Corresponding author, K. M. Meek, Structural Biophysics Research Group, School of Optometry and Vision Sciences, Cardiff University, Maindy Road, Cardiff CF24 4HQ, UK, e-mail: Meekkm@cardiff.ac.uk

**Title:**

Averaged lamellar inclination and normalised average total x-ray scatter intensity across the central and temporal aspect of the cornea and limbus

**Data List:**

Table A: X axis coordinate for the cornea (Figure 8 and Figure 10).

Table B: Y axis coordinate for the cornea (Figure 8 and Figure 10).

Table C: Averaged lamellar inclination (in degrees) across the central and temporal aspect of the cornea and limbus (Figure 8).

Table D: Average x-ray scatter across the central and temporal aspect of the cornea and limbus (Figure 10).

**Description:**

In the present paper, the geometric corneal centre has been used as an origin for the coordinate system in both Figure 8 and Figure 10. Tables A and B show the X and Y axis coordinates respectively for each plotted point in these figures. Table C displays the averaged lamellar inclination (in degrees) across the central and temporal aspect of the cornea and limbus; this data was used to create Figure 8. Table D shows the average x-ray scatter intensity across the central and temporal aspect of the cornea and limbus as shown in Figure 10.

Table A: X axis coordinate for the cornea (mm).

| 0 | 0.5 | 1 | 1.25 | 1.5 | 1.75 | 2 | 2.25 | 2.5 | 2.75 | 3 | 3.25 | 3.5 | 3.75 | 4 | 4.25 | 4.5 | 4.75 | 5 | 5.2 | 5.25 | 5.4 | 5.5 | 5.6 | 5.75 | 5.8 | 6 | 6.25 | 6.5 |
| --- | --- | --- | --- | --- | --- | --- | --- | --- | --- | --- | --- | --- | --- | --- | --- | --- | --- | --- | --- | --- | --- | --- | --- | --- | --- | --- | --- | --- |
| 0 | 0.5 | 1 | 1.25 | 1.5 | 1.75 | 2 | 2.25 | 2.5 | 2.75 | 3 | 3.25 | 3.5 | 3.75 | 4 | 4.25 | 4.5 | 4.75 | 5 | 5.2 | 5.25 | 5.4 | 5.5 | 5.6 | 5.75 | 5.8 | 6 | 6.25 | 6.5 |
| 0 | 0.5 | 1 | 1.25 | 1.5 | 1.75 | 2 | 2.25 | 2.5 | 2.75 | 3 | 3.25 | 3.5 | 3.75 | 4 | 4.25 | 4.5 | 4.75 | 5 | 5.2 | 5.25 | 5.4 | 5.5 | 5.6 | 5.75 | 5.8 | 6 | 6.25 | 6.5 |
| 0 | 0.5 | 1 | 1.25 | 1.5 | 1.75 | 2 | 2.25 | 2.5 | 2.75 | 3 | 3.25 | 3.5 | 3.75 | 4 | 4.25 | 4.5 | 4.75 | 5 | 5.2 | 5.25 | 5.4 | 5.5 | 5.6 | 5.75 | 5.8 | 6 | 6.25 | 6.5 |
| 0 | 0.5 | 1 | 1.25 | 1.5 | 1.75 | 2 | 2.25 | 2.5 | 2.75 | 3 | 3.25 | 3.5 | 3.75 | 4 | 4.25 | 4.5 | 4.75 | 5 | 5.2 | 5.25 | 5.4 | 5.5 | 5.6 | 5.75 | 5.8 | 6 | 6.25 | 6.5 |
| 0 | 0.5 | 1 | 1.25 | 1.5 | 1.75 | 2 | 2.25 | 2.5 | 2.75 | 3 | 3.25 | 3.5 | 3.75 | 4 | 4.25 | 4.5 | 4.75 | 5 | 5.2 | 5.25 | 5.4 | 5.5 | 5.6 | 5.75 | 5.8 | 6 | 6.25 | 6.5 |
| 0 | 0.5 | 1 | 1.25 | 1.5 | 1.75 | 2 | 2.25 | 2.5 | 2.75 | 3 | 3.25 | 3.5 | 3.75 | 4 | 4.25 | 4.5 | 4.75 | 5 | 5.2 | 5.25 | 5.4 | 5.5 | 5.6 | 5.75 | 5.8 | 6 | 6.25 | 6.5 |
| 0 | 0.5 | 1 | 1.25 | 1.5 | 1.75 | 2 | 2.25 | 2.5 | 2.75 | 3 | 3.25 | 3.5 | 3.75 | 4 | 4.25 | 4.5 | 4.75 | 5 | 5.2 | 5.25 | 5.4 | 5.5 | 5.6 | 5.75 | 5.8 | 6 | 6.25 | 6.5 |
| 0 | 0.5 | 1 | 1.25 | 1.5 | 1.75 | 2 | 2.25 | 2.5 | 2.75 | 3 | 3.25 | 3.5 | 3.75 | 4 | 4.25 | 4.5 | 4.75 | 5 | 5.2 | 5.25 | 5.4 | 5.5 | 5.6 | 5.75 | 5.8 | 6 | 6.25 | 6.5 |
| 0 | 0.5 | 1 | 1.25 | 1.5 | 1.75 | 2 | 2.25 | 2.5 | 2.75 | 3 | 3.25 | 3.5 | 3.75 | 4 | 4.25 | 4.5 | 4.75 | 5 | 5.2 | 5.25 | 5.4 | 5.5 | 5.6 | 5.75 | 5.8 | 6 | 6.25 | 6.5 |
| 0 | 0.5 | 1 | 1.25 | 1.5 | 1.75 | 2 | 2.25 | 2.5 | 2.75 | 3 | 3.25 | 3.5 | 3.75 | 4 | 4.25 | 4.5 | 4.75 | 5 | 5.2 | 5.25 | 5.4 | 5.5 | 5.6 | 5.75 | 5.8 | 6 | 6.25 | 6.5 |
| 0 | 0.5 | 1 | 1.25 | 1.5 | 1.75 | 2 | 2.25 | 2.5 | 2.75 | 3 | 3.25 | 3.5 | 3.75 | 4 | 4.25 | 4.5 | 4.75 | 5 | 5.2 | 5.25 | 5.4 | 5.5 | 5.6 | 5.75 | 5.8 | 6 | 6.25 | 6.5 |
| 0 | 0.5 | 1 | 1.25 | 1.5 | 1.75 | 2 | 2.25 | 2.5 | 2.75 | 3 | 3.25 | 3.5 | 3.75 | 4 | 4.25 | 4.5 | 4.75 | 5 | 5.2 | 5.25 | 5.4 | 5.5 | 5.6 | 5.75 | 5.8 | 6 | 6.25 | 6.5 |
| 0 | 0.5 | 1 | 1.25 | 1.5 | 1.75 | 2 | 2.25 | 2.5 | 2.75 | 3 | 3.25 | 3.5 | 3.75 | 4 | 4.25 | 4.5 | 4.75 | 5 | 5.2 | 5.25 | 5.4 | 5.5 | 5.6 | 5.75 | 5.8 | 6 | 6.25 | 6.5 |
| 0 | 0.5 | 1 | 1.25 | 1.5 | 1.75 | 2 | 2.25 | 2.5 | 2.75 | 3 | 3.25 | 3.5 | 3.75 | 4 | 4.25 | 4.5 | 4.75 | 5 | 5.2 | 5.25 | 5.4 | 5.5 | 5.6 | 5.75 | 5.8 | 6 | 6.25 | 6.5 |
| 0 | 0.5 | 1 | 1.25 | 1.5 | 1.75 | 2 | 2.25 | 2.5 | 2.75 | 3 | 3.25 | 3.5 | 3.75 | 4 | 4.25 | 4.5 | 4.75 | 5 | 5.2 | 5.25 | 5.4 | 5.5 | 5.6 | 5.75 | 5.8 | 6 | 6.25 | 6.5 |
| 0 | 0.5 | 1 | 1.25 | 1.5 | 1.75 | 2 | 2.25 | 2.5 | 2.75 | 3 | 3.25 | 3.5 | 3.75 | 4 | 4.25 | 4.5 | 4.75 | 5 | 5.2 | 5.25 | 5.4 | 5.5 | 5.6 | 5.75 | 5.8 | 6 | 6.25 | 6.5 |
| 0 | 0.5 | 1 | 1.25 | 1.5 | 1.75 | 2 | 2.25 | 2.5 | 2.75 | 3 | 3.25 | 3.5 | 3.75 | 4 | 4.25 | 4.5 | 4.75 | 5 | 5.2 | 5.25 | 5.4 | 5.5 | 5.6 | 5.75 | 5.8 | 6 | 6.25 | 6.5 |
| 0 | 0.5 | 1 | 1.25 | 1.5 | 1.75 | 2 | 2.25 | 2.5 | 2.75 | 3 | 3.25 | 3.5 | 3.75 | 4 | 4.25 | 4.5 | 4.75 | 5 | 5.2 | 5.25 | 5.4 | 5.5 | 5.6 | 5.75 | 5.8 | 6 | 6.25 | 6.5 |
| 0 | 0.5 | 1 | 1.25 | 1.5 | 1.75 | 2 | 2.25 | 2.5 | 2.75 | 3 | 3.25 | 3.5 | 3.75 | 4 | 4.25 | 4.5 | 4.75 | 5 | 5.2 | 5.25 | 5.4 | 5.5 | 5.6 | 5.75 | 5.8 | 6 | 6.25 | 6.5 |
| 0 | 0.5 | 1 | 1.25 | 1.5 | 1.75 | 2 | 2.25 | 2.5 | 2.75 | 3 | 3.25 | 3.5 | 3.75 | 4 | 4.25 | 4.5 | 4.75 | 5 | 5.2 | 5.25 | 5.4 | 5.5 | 5.6 | 5.75 | 5.8 | 6 | 6.25 | 6.5 |
| 0 | 0.5 | 1 | 1.25 | 1.5 | 1.75 | 2 | 2.25 | 2.5 | 2.75 | 3 | 3.25 | 3.5 | 3.75 | 4 | 4.25 | 4.5 | 4.75 | 5 | 5.2 | 5.25 | 5.4 | 5.5 | 5.6 | 5.75 | 5.8 | 6 | 6.25 | 6.5 |
| 0 | 0.5 | 1 | 1.25 | 1.5 | 1.75 | 2 | 2.25 | 2.5 | 2.75 | 3 | 3.25 | 3.5 | 3.75 | 4 | 4.25 | 4.5 | 4.75 | 5 | 5.2 | 5.25 | 5.4 | 5.5 | 5.6 | 5.75 | 5.8 | 6 | 6.25 | 6.5 |
| 0 | 0.5 | 1 | 1.25 | 1.5 | 1.75 | 2 | 2.25 | 2.5 | 2.75 | 3 | 3.25 | 3.5 | 3.75 | 4 | 4.25 | 4.5 | 4.75 | 5 | 5.2 | 5.25 | 5.4 | 5.5 | 5.6 | 5.75 | 5.8 | 6 | 6.25 | 6.5 |
| 0 | 0.5 | 1 | 1.25 | 1.5 | 1.75 | 2 | 2.25 | 2.5 | 2.75 | 3 | 3.25 | 3.5 | 3.75 | 4 | 4.25 | 4.5 | 4.75 | 5 | 5.2 | 5.25 | 5.4 | 5.5 | 5.6 | 5.75 | 5.8 | 6 | 6.25 | 6.5 |
| 0 | 0.5 | 1 | 1.25 | 1.5 | 1.75 | 2 | 2.25 | 2.5 | 2.75 | 3 | 3.25 | 3.5 | 3.75 | 4 | 4.25 | 4.5 | 4.75 | 5 | 5.2 | 5.25 | 5.4 | 5.5 | 5.6 | 5.75 | 5.8 | 6 | 6.25 | 6.5 |
| 0 | 0.5 | 1 | 1.25 | 1.5 | 1.75 | 2 | 2.25 | 2.5 | 2.75 | 3 | 3.25 | 3.5 | 3.75 | 4 | 4.25 | 4.5 | 4.75 | 5 | 5.2 | 5.25 | 5.4 | 5.5 | 5.6 | 5.75 | 5.8 | 6 | 6.25 | 6.5 |
| 0 | 0.5 | 1 | 1.25 | 1.5 | 1.75 | 2 | 2.25 | 2.5 | 2.75 | 3 | 3.25 | 3.5 | 3.75 | 4 | 4.25 | 4.5 | 4.75 | 5 | 5.2 | 5.25 | 5.4 | 5.5 | 5.6 | 5.75 | 5.8 | 6 | 6.25 | 6.5 |
| 0 | 0.5 | 1 | 1.25 | 1.5 | 1.75 | 2 | 2.25 | 2.5 | 2.75 | 3 | 3.25 | 3.5 | 3.75 | 4 | 4.25 | 4.5 | 4.75 | 5 | 5.2 | 5.25 | 5.4 | 5.5 | 5.6 | 5.75 | 5.8 | 6 | 6.25 | 6.5 |
| 0 | 0.5 | 1 | 1.25 | 1.5 | 1.75 | 2 | 2.25 | 2.5 | 2.75 | 3 | 3.25 | 3.5 | 3.75 | 4 | 4.25 | 4.5 | 4.75 | 5 | 5.2 | 5.25 | 5.4 | 5.5 | 5.6 | 5.75 | 5.8 | 6 | 6.25 | 6.5 |
| 0 | 0.5 | 1 | 1.25 | 1.5 | 1.75 | 2 | 2.25 | 2.5 | 2.75 | 3 | 3.25 | 3.5 | 3.75 | 4 | 4.25 | 4.5 | 4.75 | 5 | 5.2 | 5.25 | 5.4 | 5.5 | 5.6 | 5.75 | 5.8 | 6 | 6.25 | 6.5 |
| 0 | 0.5 | 1 | 1.25 | 1.5 | 1.75 | 2 | 2.25 | 2.5 | 2.75 | 3 | 3.25 | 3.5 | 3.75 | 4 | 4.25 | 4.5 | 4.75 | 5 | 5.2 | 5.25 | 5.4 | 5.5 | 5.6 | 5.75 | 5.8 | 6 | 6.25 | 6.5 |
| 0 | 0.5 | 1 | 1.25 | 1.5 | 1.75 | 2 | 2.25 | 2.5 | 2.75 | 3 | 3.25 | 3.5 | 3.75 | 4 | 4.25 | 4.5 | 4.75 | 5 | 5.2 | 5.25 | 5.4 | 5.5 | 5.6 | 5.75 | 5.8 | 6 | 6.25 | 6.5 |
| 0 | 0.5 | 1 | 1.25 | 1.5 | 1.75 | 2 | 2.25 | 2.5 | 2.75 | 3 | 3.25 | 3.5 | 3.75 | 4 | 4.25 | 4.5 | 4.75 | 5 | 5.2 | 5.25 | 5.4 | 5.5 | 5.6 | 5.75 | 5.8 | 6 | 6.25 | 6.5 |
| 0 | 0.5 | 1 | 1.25 | 1.5 | 1.75 | 2 | 2.25 | 2.5 | 2.75 | 3 | 3.25 | 3.5 | 3.75 | 4 | 4.25 | 4.5 | 4.75 | 5 | 5.2 | 5.25 | 5.4 | 5.5 | 5.6 | 5.75 | 5.8 | 6 | 6.25 | 6.5 |

Table B: Y axis coordinate for the cornea (mm).

| 7.3 | 7.3 | 7.2 | 7.2 | 7.1 | 7.1 | 7.0 | 6.9 | 6.8 | 6.7 | 6.6 | 6.5 | 6.3 | 6.2 | 5.9 | 5.7 | 5.5 | 5.2 | 5.0 | 4.8 | 4.8 | 4.7 | 4.6 | 4.5 | 4.4 | 4.4 | 4.3 | 4.1 | 4.0 |
| --- | --- | --- | --- | --- | --- | --- | --- | --- | --- | --- | --- | --- | --- | --- | --- | --- | --- | --- | --- | --- | --- | --- | --- | --- | --- | --- | --- | --- |
| 7.3 | 7.3 | 7.2 | 7.2 | 7.1 | 7.1 | 7.0 | 6.9 | 6.8 | 6.7 | 6.6 | 6.5 | 6.4 | 6.2 | 6.0 | 5.7 | 5.5 | 5.3 | 5.0 | 4.9 | 4.8 | 4.7 | 4.6 | 4.5 | 4.4 | 4.4 | 4.3 | 4.2 | 4.0 |
| 7.3 | 7.3 | 7.2 | 7.2 | 7.2 | 7.1 | 7.0 | 6.9 | 6.9 | 6.8 | 6.7 | 6.5 | 6.4 | 6.2 | 6.0 | 5.8 | 5.5 | 5.3 | 5.1 | 4.9 | 4.9 | 4.7 | 4.6 | 4.6 | 4.5 | 4.4 | 4.3 | 4.2 | 4.1 |
| 7.3 | 7.3 | 7.3 | 7.2 | 7.2 | 7.1 | 7.0 | 7.0 | 6.9 | 6.8 | 6.7 | 6.6 | 6.4 | 6.2 | 6.0 | 5.8 | 5.6 | 5.3 | 5.1 | 4.9 | 4.9 | 4.8 | 4.7 | 4.6 | 4.5 | 4.5 | 4.3 | 4.2 | 4.1 |
| 7.3 | 7.3 | 7.3 | 7.2 | 7.2 | 7.1 | 7.1 | 7.0 | 6.9 | 6.8 | 6.7 | 6.6 | 6.4 | 6.2 | 6.0 | 5.8 | 5.6 | 5.4 | 5.1 | 5.0 | 4.9 | 4.8 | 4.7 | 4.6 | 4.5 | 4.5 | 4.4 | 4.2 | 4.1 |
| 7.4 | 7.3 | 7.3 | 7.2 | 7.2 | 7.1 | 7.1 | 7.0 | 6.9 | 6.8 | 6.7 | 6.6 | 6.4 | 6.3 | 6.1 | 5.8 | 5.6 | 5.4 | 5.2 | 5.0 | 4.9 | 4.8 | 4.7 | 4.7 | 4.5 | 4.5 | 4.4 | 4.3 | 4.1 |
| 7.4 | 7.4 | 7.3 | 7.3 | 7.2 | 7.2 | 7.1 | 7.0 | 6.9 | 6.8 | 6.7 | 6.6 | 6.4 | 6.3 | 6.1 | 5.9 | 5.6 | 5.4 | 5.2 | 5.0 | 5.0 | 4.8 | 4.8 | 4.7 | 4.6 | 4.5 | 4.4 | 4.3 | 4.1 |
| 7.4 | 7.4 | 7.3 | 7.3 | 7.2 | 7.2 | 7.1 | 7.0 | 6.9 | 6.8 | 6.7 | 6.6 | 6.5 | 6.3 | 6.1 | 5.9 | 5.7 | 5.4 | 5.2 | 5.0 | 5.0 | 4.9 | 4.8 | 4.7 | 4.6 | 4.5 | 4.4 | 4.3 | 4.2 |
| 7.4 | 7.4 | 7.3 | 7.3 | 7.2 | 7.2 | 7.1 | 7.0 | 7.0 | 6.9 | 6.8 | 6.6 | 6.5 | 6.3 | 6.1 | 5.9 | 5.7 | 5.5 | 5.2 | 5.1 | 5.0 | 4.9 | 4.8 | 4.7 | 4.6 | 4.6 | 4.5 | 4.3 | 4.2 |
| 7.4 | 7.4 | 7.4 | 7.3 | 7.3 | 7.2 | 7.1 | 7.1 | 7.0 | 6.9 | 6.8 | 6.7 | 6.5 | 6.3 | 6.1 | 5.9 | 5.7 | 5.5 | 5.3 | 5.1 | 5.1 | 4.9 | 4.8 | 4.8 | 4.6 | 4.6 | 4.5 | 4.3 | 4.2 |
| 7.4 | 7.4 | 7.4 | 7.3 | 7.3 | 7.2 | 7.2 | 7.1 | 7.0 | 6.9 | 6.8 | 6.7 | 6.5 | 6.4 | 6.2 | 6.0 | 5.7 | 5.5 | 5.3 | 5.1 | 5.1 | 5.0 | 4.9 | 4.8 | 4.7 | 4.6 | 4.5 | 4.4 | 4.2 |
| 7.5 | 7.4 | 7.4 | 7.3 | 7.3 | 7.2 | 7.2 | 7.1 | 7.0 | 6.9 | 6.8 | 6.7 | 6.5 | 6.4 | 6.2 | 6.0 | 5.8 | 5.6 | 5.3 | 5.2 | 5.1 | 5.0 | 4.9 | 4.8 | 4.7 | 4.6 | 4.5 | 4.4 | 4.3 |
| 7.5 | 7.4 | 7.4 | 7.4 | 7.3 | 7.3 | 7.2 | 7.1 | 7.0 | 6.9 | 6.8 | 6.7 | 6.6 | 6.4 | 6.2 | 6.0 | 5.8 | 5.6 | 5.4 | 5.2 | 5.1 | 5.0 | 4.9 | 4.8 | 4.7 | 4.7 | 4.5 | 4.4 | 4.3 |
| 7.5 | 7.5 | 7.4 | 7.4 | 7.3 | 7.3 | 7.2 | 7.1 | 7.0 | 6.9 | 6.8 | 6.7 | 6.6 | 6.4 | 6.2 | 6.0 | 5.8 | 5.6 | 5.4 | 5.2 | 5.2 | 5.0 | 5.0 | 4.9 | 4.7 | 4.7 | 4.6 | 4.4 | 4.3 |
| 7.5 | 7.5 | 7.4 | 7.4 | 7.3 | 7.3 | 7.2 | 7.1 | 7.1 | 7.0 | 6.9 | 6.7 | 6.6 | 6.4 | 6.3 | 6.1 | 5.9 | 5.6 | 5.4 | 5.2 | 5.2 | 5.1 | 5.0 | 4.9 | 4.8 | 4.7 | 4.6 | 4.5 | 4.3 |
| 7.5 | 7.5 | 7.4 | 7.4 | 7.4 | 7.3 | 7.2 | 7.2 | 7.1 | 7.0 | 6.9 | 6.8 | 6.6 | 6.5 | 6.3 | 6.1 | 5.9 | 5.7 | 5.4 | 5.3 | 5.2 | 5.1 | 5.0 | 4.9 | 4.8 | 4.7 | 4.6 | 4.5 | 4.3 |
| 7.5 | 7.5 | 7.5 | 7.4 | 7.4 | 7.3 | 7.3 | 7.2 | 7.1 | 7.0 | 6.9 | 6.8 | 6.6 | 6.5 | 6.3 | 6.1 | 5.9 | 5.7 | 5.5 | 5.3 | 5.3 | 5.1 | 5.0 | 4.9 | 4.8 | 4.8 | 4.6 | 4.5 | 4.4 |
| 7.5 | 7.5 | 7.5 | 7.4 | 7.4 | 7.3 | 7.3 | 7.2 | 7.1 | 7.0 | 6.9 | 6.8 | 6.7 | 6.5 | 6.3 | 6.1 | 5.9 | 5.7 | 5.5 | 5.3 | 5.3 | 5.1 | 5.1 | 5.0 | 4.8 | 4.8 | 4.7 | 4.5 | 4.4 |
| 7.6 | 7.5 | 7.5 | 7.4 | 7.4 | 7.3 | 7.3 | 7.2 | 7.1 | 7.0 | 6.9 | 6.8 | 6.7 | 6.5 | 6.3 | 6.2 | 6.0 | 5.7 | 5.5 | 5.4 | 5.3 | 5.2 | 5.1 | 5.0 | 4.9 | 4.8 | 4.7 | 4.6 | 4.4 |
| 7.6 | 7.6 | 7.5 | 7.5 | 7.4 | 7.4 | 7.3 | 7.2 | 7.1 | 7.0 | 6.9 | 6.8 | 6.7 | 6.5 | 6.4 | 6.2 | 6.0 | 5.8 | 5.6 | 5.4 | 5.3 | 5.2 | 5.1 | 5.0 | 4.9 | 4.8 | 4.7 | 4.6 | 4.4 |
| 7.6 | 7.6 | 7.5 | 7.5 | 7.4 | 7.4 | 7.3 | 7.2 | 7.2 | 7.1 | 7.0 | 6.8 | 6.7 | 6.6 | 6.4 | 6.2 | 6.0 | 5.8 | 5.6 | 5.4 | 5.4 | 5.2 | 5.1 | 5.1 | 4.9 | 4.9 | 4.7 | 4.6 | 4.4 |
| 7.6 | 7.6 | 7.5 | 7.5 | 7.5 | 7.4 | 7.3 | 7.3 | 7.2 | 7.1 | 7.0 | 6.9 | 6.7 | 6.6 | 6.4 | 6.2 | 6.0 | 5.8 | 5.6 | 5.4 | 5.4 | 5.3 | 5.2 | 5.1 | 4.9 | 4.9 | 4.8 | 4.6 | 4.5 |
| 7.6 | 7.6 | 7.6 | 7.5 | 7.5 | 7.4 | 7.3 | 7.3 | 7.2 | 7.1 | 7.0 | 6.9 | 6.7 | 6.6 | 6.4 | 6.3 | 6.1 | 5.9 | 5.6 | 5.5 | 5.4 | 5.3 | 5.2 | 5.1 | 5.0 | 4.9 | 4.8 | 4.6 | 4.5 |
| 7.6 | 7.6 | 7.6 | 7.5 | 7.5 | 7.4 | 7.4 | 7.3 | 7.2 | 7.1 | 7.0 | 6.9 | 6.8 | 6.6 | 6.5 | 6.3 | 6.1 | 5.9 | 5.7 | 5.5 | 5.5 | 5.3 | 5.2 | 5.1 | 5.0 | 4.9 | 4.8 | 4.7 | 4.5 |
| 7.6 | 7.6 | 7.6 | 7.5 | 7.5 | 7.4 | 7.4 | 7.3 | 7.2 | 7.1 | 7.0 | 6.9 | 6.8 | 6.6 | 6.5 | 6.3 | 6.1 | 5.9 | 5.7 | 5.5 | 5.5 | 5.3 | 5.3 | 5.2 | 5.0 | 5.0 | 4.8 | 4.7 | 4.5 |
| 7.7 | 7.6 | 7.6 | 7.6 | 7.5 | 7.5 | 7.4 | 7.3 | 7.2 | 7.1 | 7.0 | 6.9 | 6.8 | 6.7 | 6.5 | 6.3 | 6.1 | 5.9 | 5.7 | 5.6 | 5.5 | 5.4 | 5.3 | 5.2 | 5.0 | 5.0 | 4.9 | 4.7 | 4.6 |
| 7.7 | 7.7 | 7.6 | 7.6 | 7.5 | 7.5 | 7.4 | 7.3 | 7.3 | 7.2 | 7.1 | 6.9 | 6.8 | 6.7 | 6.5 | 6.3 | 6.2 | 6.0 | 5.8 | 5.6 | 5.5 | 5.4 | 5.3 | 5.2 | 5.1 | 5.0 | 4.9 | 4.7 | 4.6 |
| 7.7 | 7.7 | 7.6 | 7.6 | 7.5 | 7.5 | 7.4 | 7.4 | 7.3 | 7.2 | 7.1 | 7.0 | 6.8 | 6.7 | 6.5 | 6.4 | 6.2 | 6.0 | 5.8 | 5.6 | 5.6 | 5.4 | 5.3 | 5.2 | 5.1 | 5.0 | 4.9 | 4.8 | 4.6 |
| 7.7 | 7.7 | 7.6 | 7.6 | 7.6 | 7.5 | 7.4 | 7.4 | 7.3 | 7.2 | 7.1 | 7.0 | 6.9 | 6.7 | 6.6 | 6.4 | 6.2 | 6.0 | 5.8 | 5.6 | 5.6 | 5.5 | 5.4 | 5.3 | 5.1 | 5.1 | 4.9 | 4.8 | 4.6 |
| 7.7 | 7.7 | 7.7 | 7.6 | 7.6 | 7.5 | 7.5 | 7.4 | 7.3 | 7.2 | 7.1 | 7.0 | 6.9 | 6.7 | 6.6 | 6.4 | 6.2 | 6.0 | 5.8 | 5.7 | 5.6 | 5.5 | 5.4 | 5.3 | 5.1 | 5.1 | 5.0 | 4.8 | 4.6 |
| 7.7 | 7.7 | 7.7 | 7.6 | 7.6 | 7.5 | 7.5 | 7.4 | 7.3 | 7.2 | 7.1 | 7.0 | 6.9 | 6.8 | 6.6 | 6.4 | 6.3 | 6.1 | 5.9 | 5.7 | 5.7 | 5.5 | 5.4 | 5.3 | 5.2 | 5.1 | 5.0 | 4.8 | 4.7 |
| 7.8 | 7.7 | 7.7 | 7.7 | 7.6 | 7.6 | 7.5 | 7.4 | 7.3 | 7.2 | 7.1 | 7.0 | 6.9 | 6.8 | 6.6 | 6.5 | 6.3 | 6.1 | 5.9 | 5.7 | 5.7 | 5.5 | 5.4 | 5.3 | 5.2 | 5.1 | 5.0 | 4.8 | 4.7 |
| 7.8 | 7.8 | 7.7 | 7.7 | 7.6 | 7.6 | 7.5 | 7.4 | 7.4 | 7.3 | 7.2 | 7.1 | 6.9 | 6.8 | 6.7 | 6.5 | 6.3 | 6.1 | 5.9 | 5.8 | 5.7 | 5.6 | 5.5 | 5.4 | 5.2 | 5.2 | 5.0 | 4.9 | 4.7 |
| 7.8 | 7.8 | 7.7 | 7.7 | 7.6 | 7.6 | 7.5 | 7.5 | 7.4 | 7.3 | 7.2 | 7.1 | 6.9 | 6.8 | 6.7 | 6.5 | 6.3 | 6.2 | 6.0 | 5.8 | 5.7 | 5.6 | 5.5 | 5.4 | 5.2 | 5.2 | 5.0 | 4.9 | 4.7 |
| 7.8 | 7.8 | 7.7 | 7.7 | 7.7 | 7.6 | 7.5 | 7.5 | 7.4 | 7.3 | 7.2 | 7.1 | 7.0 | 6.8 | 6.7 | 6.5 | 6.4 | 6.2 | 6.0 | 5.8 | 5.8 | 5.6 | 5.5 | 5.4 | 5.3 | 5.2 | 5.1 | 4.9 | 4.7 |

Table C: Averaged lamellar inclination (in degrees) across the central and temporal aspect of the cornea and limbus.

| 12.0 | 9.7 | 8.1 | 11.6 | 10.6 | 16.7 | 10.6 | 9.7 | 9.1 | 17.3 | 11.2 | 7.9 | 8.9 | 8.7 | 8.9 | 7.7 | 9.5 | 7.4 | 9.0 | 9.9 | 8.2 | 10.8 | 12.8 | 7.2 | 15.7 | 8.7 | 11.7 | 10.7 | 10.2 | 9.7 |
| --- | --- | --- | --- | --- | --- | --- | --- | --- | --- | --- | --- | --- | --- | --- | --- | --- | --- | --- | --- | --- | --- | --- | --- | --- | --- | --- | --- | --- | --- |
| 8.6 | 8.2 | 7.4 | 10.1 | 8.7 | 12.8 | 8.9 | 9.1 | 8.3 | 11.3 | 9.7 | 7.9 | 9.3 | 8.2 | 9.3 | 8.0 | 10.1 | 7.4 | 9.4 | 10.1 | 8.1 | 9.0 | 12.3 | 8.6 | 11.5 | 9.0 | 13.5 | 10.4 | 10.7 | 9.6 |
| 7.8 | 8.0 | 7.3 | 9.5 | 7.9 | 10.8 | 8.2 | 9.1 | 7.7 | 8.9 | 9.5 | 7.9 | 8.9 | 7.8 | 9.0 | 8.0 | 10.2 | 7.4 | 9.4 | 9.6 | 8.0 | 9.0 | 12.9 | 8.8 | 8.4 | 8.8 | 13.3 | 10.5 | 10.8 | 10.3 |
| 8.0 | 8.0 | 7.3 | 9.5 | 7.7 | 10.0 | 8.0 | 9.2 | 7.5 | 8.4 | 9.5 | 8.1 | 8.5 | 7.6 | 9.1 | 7.7 | 10.3 | 7.5 | 9.5 | 9.3 | 8.2 | 9.2 | 13.5 | 9.3 | 8.6 | 9.1 | 13.4 | 10.8 | 10.2 | 11.0 |
| 7.9 | 8.0 | 7.4 | 9.5 | 7.5 | 9.5 | 8.1 | 9.1 | 7.6 | 8.2 | 9.3 | 7.8 | 8.5 | 7.5 | 9.3 | 7.9 | 10.4 | 7.6 | 9.6 | 8.9 | 8.4 | 9.5 | 12.9 | 9.3 | 8.6 | 9.6 | 12.8 | 10.9 | 9.2 | 11.0 |
| 7.7 | 8.1 | 7.5 | 9.8 | 7.7 | 9.1 | 8.2 | 9.0 | 7.8 | 8.1 | 9.1 | 7.6 | 8.6 | 7.5 | 9.2 | 7.8 | 10.6 | 7.7 | 9.4 | 8.9 | 8.3 | 9.4 | 10.3 | 9.5 | 8.6 | 9.9 | 13.6 | 10.9 | 9.7 | 11.0 |
| 7.8 | 8.1 | 7.6 | 10.1 | 7.8 | 8.8 | 8.2 | 9.0 | 7.9 | 8.2 | 8.9 | 7.5 | 8.7 | 7.6 | 9.0 | 7.6 | 10.8 | 7.7 | 9.7 | 8.6 | 8.2 | 9.5 | 9.7 | 9.5 | 8.5 | 10.1 | 14.0 | 11.3 | 8.5 | 8.1 |
| 7.9 | 8.1 | 7.7 | 10.0 | 7.7 | 8.6 | 8.5 | 8.9 | 8.0 | 8.4 | 8.8 | 7.5 | 8.7 | 7.8 | 9.1 | 7.3 | 10.8 | 7.8 | 9.5 | 8.6 | 8.1 | 9.7 | 9.4 | 9.8 | 8.5 | 9.9 | 10.1 | 11.7 | 8.7 | 6.4 |
| 7.9 | 8.3 | 7.6 | 9.4 | 7.7 | 8.5 | 8.6 | 8.8 | 7.9 | 8.5 | 8.7 | 7.5 | 8.6 | 8.0 | 9.3 | 7.3 | 10.8 | 7.8 | 9.5 | 8.8 | 8.1 | 9.5 | 9.7 | 9.7 | 8.3 | 9.8 | 10.5 | 11.7 | 11.0 | 9.9 |
| 7.9 | 8.4 | 7.8 | 9.1 | 7.8 | 8.7 | 8.4 | 8.7 | 7.9 | 8.6 | 8.4 | 7.4 | 8.6 | 8.3 | 9.3 | 7.4 | 10.7 | 7.8 | 9.4 | 8.8 | 8.0 | 9.6 | 9.9 | 9.7 | 8.2 | 9.8 | 10.4 | 11.8 | 12.1 | 10.1 |
| 8.1 | 8.4 | 7.8 | 9.2 | 7.7 | 8.8 | 8.5 | 8.6 | 7.7 | 8.6 | 8.5 | 7.5 | 8.7 | 8.4 | 9.3 | 7.4 | 10.7 | 7.6 | 9.3 | 8.3 | 8.0 | 9.4 | 10.0 | 9.7 | 8.2 | 9.7 | 9.9 | 12.3 | 12.8 | 10.3 |
| 8.1 | 8.4 | 7.8 | 9.3 | 7.7 | 8.8 | 8.5 | 8.8 | 7.8 | 8.6 | 8.5 | 7.7 | 8.8 | 8.3 | 9.3 | 7.4 | 10.7 | 7.5 | 9.5 | 8.2 | 8.0 | 9.1 | 10.1 | 9.7 | 8.2 | 9.7 | 9.3 | 12.2 | 12.1 | 10.4 |
| 8.1 | 8.3 | 7.8 | 9.2 | 7.7 | 8.9 | 8.4 | 9.0 | 7.8 | 8.7 | 8.6 | 7.6 | 8.7 | 8.3 | 9.4 | 7.4 | 10.6 | 7.6 | 9.4 | 8.2 | 8.0 | 9.2 | 10.2 | 9.5 | 8.2 | 9.8 | 9.5 | 12.1 | 12.5 | 10.3 |
| 8.3 | 8.3 | 8.0 | 9.1 | 7.7 | 8.9 | 8.3 | 9.0 | 7.8 | 8.7 | 8.6 | 6.7 | 8.7 | 8.0 | 9.3 | 7.3 | 10.5 | 7.8 | 9.3 | 8.0 | 8.0 | 8.9 | 10.6 | 9.3 | 8.4 | 9.8 | 9.8 | 11.9 | 12.3 | 10.0 |
| 8.3 | 8.3 | 8.0 | 9.1 | 7.7 | 9.0 | 8.4 | 9.0 | 7.8 | 8.8 | 8.6 | 5.2 | 8.7 | 8.3 | 9.1 | 7.4 | 10.5 | 8.0 | 9.8 | 8.1 | 8.1 | 8.7 | 10.5 | 9.3 | 8.7 | 9.9 | 9.6 | 11.2 | 11.4 | 10.0 |
| 8.4 | 8.3 | 7.9 | 9.4 | 7.9 | 9.1 | 8.3 | 8.9 | 7.8 | 8.9 | 8.7 | 6.6 | 8.7 | 8.7 | 9.1 | 7.4 | 10.7 | 8.1 | 10.1 | 8.1 | 8.1 | 8.7 | 9.8 | 9.3 | 8.5 | 9.9 | 9.7 | 10.4 | 11.2 | 9.3 |
| 8.5 | 8.4 | 8.1 | 9.6 | 8.1 | 9.1 | 8.3 | 9.1 | 7.9 | 8.9 | 8.8 | 7.5 | 8.7 | 8.5 | 9.1 | 7.4 | 10.8 | 8.2 | 10.3 | 8.3 | 8.2 | 8.6 | 9.5 | 9.3 | 8.4 | 10.0 | 9.5 | 9.9 | 11.1 | 7.2 |
| 8.4 | 8.5 | 8.2 | 9.5 | 8.2 | 9.0 | 8.3 | 9.1 | 8.1 | 9.0 | 8.7 | 7.6 | 8.7 | 8.3 | 9.0 | 7.5 | 10.8 | 8.1 | 10.4 | 8.3 | 8.2 | 8.6 | 9.7 | 9.3 | 8.4 | 9.6 | 9.5 | 9.7 | 11.3 | 9.2 |
| 8.5 | 8.5 | 8.4 | 9.4 | 8.2 | 8.8 | 8.6 | 9.1 | 8.3 | 9.0 | 8.7 | 7.7 | 8.7 | 8.2 | 9.1 | 7.6 | 11.9 | 8.1 | 10.4 | 8.4 | 8.2 | 8.9 | 10.0 | 9.6 | 8.5 | 9.9 | 9.5 | 9.3 | 10.7 | 9.7 |
| 8.5 | 8.5 | 8.4 | 9.3 | 8.3 | 8.7 | 8.4 | 9.1 | 8.4 | 9.0 | 8.8 | 7.8 | 8.7 | 7.7 | 9.1 | 7.8 | 12.9 | 8.2 | 11.5 | 8.7 | 8.2 | 8.9 | 10.3 | 9.7 | 8.6 | 10.1 | 9.2 | 6.7 | 11.5 | 9.5 |
| 8.6 | 8.6 | 8.5 | 9.1 | 8.6 | 8.8 | 8.4 | 8.9 | 8.5 | 8.9 | 9.1 | 7.9 | 8.7 | 6.3 | 9.2 | 7.8 | 12.7 | 8.2 | 11.5 | 8.8 | 8.2 | 9.1 | 10.0 | 9.7 | 8.5 | 10.3 | 8.7 | 9.5 | 11.4 | 9.4 |
| 8.8 | 8.8 | 8.7 | 9.3 | 8.7 | 8.9 | 8.5 | 8.9 | 8.5 | 8.8 | 9.0 | 7.9 | 8.8 | 7.8 | 9.3 | 7.8 | 12.9 | 8.2 | 11.6 | 9.0 | 8.3 | 9.6 | 9.9 | 10.1 | 8.4 | 10.5 | 9.4 | 9.9 | 11.6 | 9.4 |
| 9.2 | 8.9 | 8.9 | 9.6 | 8.7 | 9.0 | 8.7 | 9.1 | 8.5 | 8.8 | 9.1 | 7.9 | 8.9 | 8.4 | 9.5 | 7.8 | 12.4 | 8.2 | 11.4 | 9.1 | 8.4 | 9.9 | 10.1 | 9.9 | 8.4 | 10.4 | 9.9 | 9.9 | 11.7 | 9.5 |
| 9.5 | 9.1 | 9.1 | 9.8 | 9.0 | 9.2 | 8.9 | 9.2 | 8.8 | 9.0 | 9.3 | 8.0 | 9.0 | 8.6 | 9.6 | 8.0 | 12.1 | 8.2 | 10.5 | 9.2 | 8.6 | 9.9 | 10.0 | 9.9 | 8.5 | 10.6 | 9.5 | 9.9 | 11.2 | 9.6 |
| 9.6 | 9.2 | 9.3 | 9.8 | 9.2 | 9.3 | 8.9 | 9.2 | 9.2 | 9.1 | 9.4 | 8.0 | 9.1 | 8.9 | 9.7 | 8.2 | 12.5 | 8.3 | 9.6 | 9.5 | 8.8 | 9.7 | 9.7 | 10.1 | 8.7 | 10.6 | 9.5 | 9.7 | 11.1 | 9.6 |
| 9.7 | 9.3 | 9.4 | 9.8 | 9.4 | 9.3 | 9.2 | 9.3 | 9.6 | 9.1 | 9.5 | 8.1 | 9.2 | 11.2 | 9.7 | 8.5 | 13.3 | 8.5 | 9.2 | 9.8 | 8.9 | 10.3 | 9.8 | 10.0 | 8.8 | 10.6 | 9.6 | 9.5 | 11.1 | 9.3 |
| 10.0 | 9.4 | 9.7 | 9.8 | 9.5 | 9.3 | 9.3 | 9.4 | 9.7 | 9.3 | 9.7 | 8.4 | 9.3 | 10.6 | 9.7 | 8.7 | 13.9 | 8.9 | 9.5 | 10.3 | 9.0 | 10.5 | 9.9 | 10.1 | 8.8 | 10.4 | 9.7 | 9.4 | 11.1 | 7.4 |
| 10.1 | 9.8 | 9.8 | 9.9 | 9.6 | 9.4 | 9.4 | 9.5 | 9.8 | 9.5 | 9.8 | 8.6 | 9.3 | 11.2 | 9.8 | 8.9 | 12.9 | 9.2 | 10.1 | 10.7 | 9.0 | 10.6 | 9.7 | 10.4 | 8.0 | 10.3 | 9.4 | 9.5 | 11.3 | 6.4 |
| 10.2 | 10.0 | 9.9 | 10.1 | 10.1 | 9.5 | 9.6 | 9.5 | 9.8 | 9.5 | 9.8 | 8.8 | 9.0 | 17.2 | 10.1 | 9.1 | 13.1 | 9.5 | 9.9 | 11.1 | 9.0 | 10.7 | 9.5 | 10.6 | 7.0 | 10.7 | 9.4 | 9.7 | 11.3 | 9.0 |
| 10.6 | 10.3 | 10.6 | 10.8 | 10.3 | 9.9 | 10.2 | 9.8 | 9.9 | 9.8 | 10.6 | 9.1 | 9.9 | 10.0 | 10.5 | 9.4 | 12.9 | 9.8 | 8.1 | 11.0 | 10.7 | 11.3 | 10.0 | 10.8 | 9.3 | 10.9 | 9.6 | 9.7 | 11.5 | 9.3 |
| 10.8 | 10.8 | 10.9 | 10.7 | 10.7 | 10.2 | 10.5 | 10.0 | 10.1 | 9.9 | 10.7 | 9.5 | 10.3 | 10.3 | 11.2 | 9.6 | 13.1 | 10.4 | 9.8 | 11.0 | 9.6 | 11.9 | 9.6 | 11.0 | 9.7 | 10.9 | 9.6 | 9.7 | 11.0 | 9.4 |
| 10.9 | 10.9 | 10.9 | 11.0 | 10.8 | 10.4 | 10.8 | 10.2 | 10.5 | 10.2 | 10.9 | 9.6 | 10.5 | 10.6 | 11.4 | 10.1 | 12.9 | 10.4 | 9.8 | 11.0 | 10.1 | 12.2 | 10.0 | 11.0 | 9.8 | 10.8 | 9.8 | 9.9 | 10.6 | 9.8 |
| 10.7 | 10.8 | 11.2 | 12.4 | 11.0 | 10.6 | 10.2 | 10.4 | 10.6 | 10.6 | 11.2 | 10.0 | 11.1 | 10.6 | 11.3 | 10.6 | 13.2 | 10.7 | 10.4 | 10.8 | 10.4 | 11.7 | 10.2 | 11.0 | 9.9 | 10.8 | 10.0 | 10.2 | 10.1 | 11.9 |
| 10.9 | 11.3 | 11.7 | 15.2 | 11.9 | 13.7 | 10.9 | 10.6 | 10.1 | 12.9 | 11.2 | 10.6 | 11.6 | 11.0 | 11.4 | 10.3 | 12.9 | 11.1 | 10.6 | 11.6 | 10.8 | 12.7 | 10.4 | 12.2 | 10.1 | 10.8 | 10.4 | 10.7 | 10.1 | 13.0 |
| 16.2 | 12.4 | 12.9 | 18.8 | 13.4 | 18.0 | 10.3 | 10.9 | 14.9 | 16.1 | 10.9 | 10.7 | 13.5 | 8.5 | 11.0 | 11.6 | 12.7 | 11.3 | 11.6 | 15.0 | 10.9 | 14.3 | 10.2 | 14.1 | 10.3 | 10.8 | 10.6 | 10.8 | 10.3 | 14.4 |

Table D: Average total x-ray scatter across the central and temporal aspect of the cornea and limbus (arbitrary units).

| 1.8 | 1.8 | 3.9 | 2.9 | 1.7 | 3.3 | 1.9 | 3.4 | 2.2 | 3.5 | 3.0 | 18.7 | 9.9 | 3.7 | 16.5 | 3.9 | 31.5 | 3.9 | 3.4 | 2.3 | 1.4 | 1.6 | 0.9 | 1.5 | 1.0 | 2.0 | 2.4 | 2.5 | 4.4 |
| --- | --- | --- | --- | --- | --- | --- | --- | --- | --- | --- | --- | --- | --- | --- | --- | --- | --- | --- | --- | --- | --- | --- | --- | --- | --- | --- | --- | --- |
| 2.2 | 3.3 | 5.0 | 7.0 | 2.9 | 3.9 | 3.9 | 6.1 | 4.0 | 3.6 | 4.6 | 19.7 | 12.5 | 7.1 | 20.2 | 5.4 | 33.1 | 7.8 | 8.5 | 5.2 | 3.9 | 7.1 | 13.3 | 3.1 | 1.4 | 3.1 | 7.1 | 4.8 | 12.5 |
| 3.7 | 5.9 | 6.9 | 10.4 | 6.1 | 4.8 | 7.7 | 8.0 | 7.4 | 5.7 | 7.3 | 20.6 | 15.5 | 10.5 | 24.0 | 6.8 | 37.7 | 11.6 | 14.5 | 11.1 | 6.4 | 17.9 | 16.1 | 13.3 | 2.9 | 10.0 | 17.2 | 7.7 | 17.4 |
| 6.2 | 9.0 | 9.9 | 12.9 | 8.8 | 6.2 | 12.1 | 9.3 | 11.9 | 9.1 | 11.0 | 21.2 | 19.2 | 13.4 | 27.1 | 8.1 | 43.0 | 15.5 | 21.0 | 20.1 | 11.9 | 28.0 | 19.0 | 33.0 | 7.8 | 29.7 | 28.5 | 10.5 | 20.0 |
| 9.0 | 11.3 | 14.1 | 13.9 | 11.9 | 7.6 | 15.2 | 10.1 | 15.1 | 13.1 | 15.5 | 21.1 | 24.0 | 15.5 | 29.6 | 10.5 | 47.6 | 17.8 | 26.7 | 30.1 | 17.4 | 43.5 | 21.4 | 51.1 | 13.4 | 58.5 | 33.9 | 12.9 | 19.5 |
| 12.6 | 13.4 | 16.9 | 14.8 | 13.8 | 9.5 | 16.4 | 10.9 | 17.8 | 16.9 | 18.6 | 21.0 | 29.3 | 18.0 | 30.8 | 13.0 | 51.9 | 18.4 | 31.8 | 38.2 | 23.0 | 57.1 | 27.1 | 63.9 | 19.6 | 71.2 | 37.6 | 14.7 | 21.6 |
| 15.6 | 14.7 | 18.2 | 16.4 | 15.8 | 12.1 | 17.8 | 12.0 | 20.2 | 20.7 | 20.3 | 21.2 | 33.7 | 20.4 | 32.3 | 15.7 | 54.2 | 20.0 | 35.9 | 48.7 | 28.5 | 62.0 | 31.0 | 71.5 | 26.0 | 71.5 | 39.0 | 16.1 | 23.0 |
| 16.7 | 16.4 | 19.1 | 18.0 | 17.4 | 15.3 | 19.3 | 13.7 | 21.8 | 24.9 | 22.2 | 22.7 | 36.9 | 22.5 | 35.0 | 18.3 | 54.2 | 21.9 | 40.1 | 53.3 | 33.0 | 61.3 | 33.6 | 70.8 | 32.3 | 72.4 | 41.3 | 17.0 | 20.6 |
| 17.5 | 17.3 | 19.5 | 20.0 | 18.9 | 18.8 | 21.0 | 15.9 | 24.2 | 29.6 | 24.3 | 24.3 | 37.6 | 24.4 | 37.3 | 20.6 | 52.0 | 24.4 | 43.2 | 55.3 | 37.4 | 58.3 | 34.1 | 63.5 | 36.3 | 70.3 | 40.9 | 17.5 | 25.9 |
| 17.7 | 17.3 | 19.8 | 24.5 | 20.0 | 20.8 | 22.5 | 19.1 | 25.6 | 34.7 | 25.5 | 25.1 | 38.3 | 25.3 | 38.6 | 23.0 | 49.7 | 27.5 | 45.1 | 56.1 | 39.7 | 55.5 | 33.7 | 59.3 | 40.3 | 62.8 | 40.9 | 18.6 | 27.9 |
| 18.7 | 17.5 | 20.2 | 29.4 | 21.1 | 24.0 | 23.4 | 25.5 | 26.7 | 39.2 | 27.1 | 25.9 | 38.7 | 25.7 | 39.1 | 26.0 | 48.4 | 32.4 | 47.1 | 53.8 | 42.0 | 52.3 | 35.0 | 58.8 | 44.0 | 60.4 | 41.0 | 19.5 | 27.8 |
| 19.2 | 18.1 | 20.4 | 33.3 | 21.8 | 28.0 | 25.1 | 30.0 | 29.2 | 41.4 | 28.6 | 26.7 | 38.3 | 26.5 | 39.8 | 28.4 | 48.0 | 36.7 | 47.8 | 51.5 | 44.2 | 49.7 | 37.5 | 55.6 | 46.2 | 58.3 | 41.6 | 20.7 | 28.5 |
| 20.3 | 18.5 | 21.5 | 34.1 | 23.7 | 32.6 | 25.5 | 33.1 | 30.0 | 42.5 | 30.5 | 28.4 | 37.7 | 28.9 | 40.1 | 30.5 | 48.6 | 40.2 | 47.6 | 48.5 | 46.4 | 50.2 | 38.6 | 51.5 | 46.9 | 54.0 | 41.6 | 23.7 | 29.9 |
| 20.8 | 19.3 | 22.5 | 34.0 | 25.4 | 34.6 | 26.2 | 35.0 | 30.7 | 42.7 | 31.7 | 28.3 | 37.4 | 31.7 | 40.5 | 33.0 | 48.3 | 41.9 | 47.2 | 47.0 | 46.4 | 46.6 | 39.1 | 48.5 | 45.0 | 50.3 | 40.7 | 30.8 | 30.8 |
| 21.3 | 20.0 | 22.8 | 34.0 | 26.1 | 35.2 | 27.3 | 35.8 | 31.8 | 42.1 | 32.8 | 26.9 | 37.6 | 33.3 | 41.7 | 35.0 | 47.9 | 43.0 | 47.7 | 46.6 | 46.3 | 44.7 | 39.9 | 47.8 | 42.2 | 49.3 | 41.0 | 36.8 | 30.3 |
| 21.5 | 20.1 | 23.3 | 34.3 | 26.3 | 35.7 | 28.4 | 36.5 | 32.5 | 41.0 | 33.8 | 30.6 | 38.0 | 34.1 | 41.8 | 36.8 | 47.4 | 44.1 | 48.8 | 46.7 | 46.1 | 43.9 | 40.3 | 47.1 | 45.0 | 48.1 | 40.1 | 38.4 | 29.7 |
| 21.1 | 20.2 | 23.9 | 34.5 | 26.8 | 36.2 | 27.5 | 37.6 | 32.5 | 40.4 | 34.4 | 32.2 | 38.1 | 34.5 | 41.3 | 38.2 | 46.6 | 44.9 | 48.8 | 44.4 | 45.9 | 42.5 | 41.2 | 45.5 | 47.1 | 45.9 | 39.6 | 38.2 | 30.3 |
| 21.1 | 21.4 | 24.2 | 34.3 | 26.6 | 36.2 | 27.4 | 38.3 | 32.1 | 41.0 | 33.9 | 31.1 | 38.4 | 35.0 | 40.2 | 37.5 | 46.2 | 44.8 | 48.2 | 42.9 | 46.4 | 41.6 | 41.3 | 43.9 | 47.1 | 45.9 | 40.1 | 40.0 | 30.1 |
| 22.4 | 21.6 | 24.0 | 33.9 | 26.2 | 35.8 | 27.7 | 38.1 | 32.7 | 42.3 | 33.5 | 31.9 | 38.9 | 34.7 | 38.5 | 37.0 | 44.6 | 44.5 | 47.9 | 40.6 | 46.9 | 43.4 | 41.7 | 45.0 | 46.3 | 45.2 | 40.6 | 40.9 | 27.6 |
| 23.3 | 21.7 | 23.6 | 33.0 | 25.9 | 35.4 | 27.7 | 36.0 | 31.5 | 42.0 | 33.9 | 32.6 | 39.1 | 33.5 | 37.4 | 38.1 | 43.1 | 43.8 | 46.1 | 41.0 | 46.1 | 43.0 | 41.8 | 44.3 | 44.7 | 45.0 | 40.4 | 41.8 | 30.5 |
| 23.2 | 21.6 | 23.4 | 31.5 | 24.7 | 35.4 | 27.6 | 35.5 | 32.3 | 40.7 | 35.1 | 32.5 | 39.5 | 31.3 | 37.0 | 37.8 | 42.2 | 43.2 | 44.2 | 38.9 | 45.2 | 41.0 | 40.4 | 42.4 | 44.2 | 43.0 | 38.6 | 42.4 | 30.6 |
| 23.1 | 21.4 | 22.6 | 31.6 | 24.7 | 34.9 | 27.5 | 35.2 | 32.0 | 40.2 | 35.8 | 32.6 | 39.5 | 32.8 | 37.6 | 37.2 | 41.4 | 42.5 | 41.5 | 37.1 | 46.3 | 39.5 | 40.0 | 40.7 | 44.1 | 39.0 | 37.3 | 40.5 | 31.8 |
| 24.0 | 20.8 | 22.9 | 32.2 | 24.9 | 33.5 | 27.4 | 35.0 | 31.2 | 40.0 | 36.1 | 32.7 | 39.0 | 33.0 | 37.9 | 36.3 | 41.1 | 40.1 | 39.5 | 34.6 | 47.5 | 37.7 | 39.7 | 35.3 | 44.6 | 36.4 | 36.6 | 40.7 | 30.0 |
| 24.9 | 20.8 | 23.4 | 33.0 | 23.8 | 32.1 | 26.9 | 35.2 | 30.5 | 39.1 | 35.7 | 32.6 | 38.5 | 32.6 | 38.6 | 35.5 | 39.0 | 38.4 | 37.6 | 31.0 | 46.5 | 32.5 | 39.0 | 32.5 | 45.1 | 33.6 | 36.0 | 43.5 | 30.8 |
| 25.0 | 20.8 | 23.1 | 33.9 | 23.8 | 33.4 | 26.2 | 34.3 | 30.0 | 37.2 | 35.0 | 31.7 | 38.0 | 32.7 | 38.2 | 35.0 | 37.5 | 38.4 | 36.2 | 27.9 | 45.5 | 26.6 | 38.3 | 29.1 | 45.2 | 30.2 | 34.3 | 44.6 | 31.0 |
| 24.7 | 20.6 | 22.8 | 33.4 | 23.5 | 35.6 | 25.6 | 32.6 | 30.1 | 35.6 | 34.7 | 30.6 | 38.0 | 25.5 | 37.5 | 34.9 | 35.9 | 38.4 | 33.8 | 26.1 | 44.1 | 22.7 | 36.4 | 24.2 | 45.1 | 25.0 | 31.9 | 43.6 | 33.3 |
| 24.2 | 21.0 | 22.6 | 30.6 | 24.7 | 34.1 | 24.7 | 32.1 | 29.6 | 35.8 | 34.0 | 30.2 | 38.0 | 26.1 | 37.0 | 34.8 | 34.8 | 38.5 | 32.1 | 23.4 | 42.6 | 19.6 | 34.4 | 19.0 | 44.7 | 18.3 | 27.5 | 43.4 | 31.0 |
| 24.2 | 21.7 | 21.9 | 28.4 | 25.4 | 32.2 | 23.7 | 32.0 | 29.0 | 35.3 | 33.6 | 29.2 | 37.3 | 26.8 | 34.9 | 34.1 | 34.0 | 36.3 | 27.9 | 20.4 | 42.3 | 17.9 | 32.9 | 17.3 | 42.8 | 12.7 | 24.0 | 42.3 | 28.0 |
| 24.4 | 21.3 | 21.3 | 28.1 | 25.5 | 31.3 | 22.2 | 31.6 | 28.2 | 30.9 | 33.8 | 27.7 | 35.4 | 19.5 | 32.6 | 32.7 | 31.8 | 33.2 | 23.7 | 16.0 | 42.0 | 14.7 | 31.1 | 14.3 | 40.3 | 10.6 | 22.5 | 43.9 | 28.9 |
| 23.9 | 20.1 | 20.0 | 25.8 | 24.7 | 29.4 | 20.6 | 28.0 | 26.9 | 28.4 | 32.8 | 25.9 | 36.9 | 26.2 | 31.5 | 31.1 | 30.9 | 30.6 | 20.5 | 12.3 | 35.8 | 10.7 | 28.6 | 11.2 | 41.2 | 8.3 | 20.5 | 42.8 | 29.9 |
| 21.6 | 18.9 | 18.3 | 23.7 | 21.3 | 27.8 | 19.0 | 25.8 | 24.3 | 23.6 | 33.2 | 25.4 | 34.1 | 25.7 | 28.3 | 27.8 | 29.0 | 27.9 | 20.1 | 10.2 | 38.2 | 7.5 | 26.8 | 8.4 | 39.8 | 7.5 | 19.2 | 42.4 | 28.5 |
| 16.8 | 16.2 | 14.7 | 19.2 | 18.5 | 23.0 | 16.9 | 23.1 | 20.3 | 17.7 | 30.6 | 23.6 | 31.5 | 21.4 | 24.8 | 26.9 | 25.4 | 27.9 | 16.4 | 8.4 | 35.8 | 4.9 | 24.7 | 5.8 | 35.3 | 6.8 | 18.5 | 39.9 | 27.3 |
| 12.6 | 12.2 | 9.3 | 11.5 | 13.0 | 15.5 | 13.3 | 19.2 | 13.5 | 9.5 | 24.6 | 19.9 | 25.8 | 17.1 | 18.8 | 23.6 | 21.5 | 24.5 | 12.5 | 6.1 | 31.8 | 3.1 | 21.4 | 3.4 | 29.0 | 6.1 | 15.3 | 35.6 | 26.1 |
| 9.2 | 8.2 | 4.6 | 3.7 | 6.1 | 3.8 | 6.4 | 12.5 | 4.5 | 5.6 | 16.2 | 15.4 | 13.9 | 4.7 | 10.5 | 19.4 | 16.2 | 21.8 | 9.3 | 3.7 | 27.6 | 2.0 | 12.9 | 2.1 | 20.7 | 5.4 | 10.9 | 28.5 | 24.0 |
| 7.2 | 4.3 | 1.7 | 3.4 | 1.6 | 3.7 | 2.1 | 4.4 | 2.2 | 3.8 | 7.1 | 11.9 | 7.8 | 0.7 | 2.2 | 10.0 | 8.0 | 13.3 | 5.3 | 2.0 | 18.6 | 1.4 | 4.6 | 1.3 | 8.2 | 4.6 | 4.8 | 12.3 | 20.6 |
